# Xanthone Dimers in Angiosperms, Fungi, Lichens: Comprehensive Review of Their Sources, Structures, and Pharmacological Properties

**DOI:** 10.3390/molecules30040967

**Published:** 2025-02-19

**Authors:** Fengzhi Shi, Min Fan, Haifeng Li, Shiwei Li, Shuang Wang

**Affiliations:** 1 College of Pharmacy, Dali University, Dali 671000, China; 18487540229@163.com (F.S.); fanmin302301@163.com (M.F.); lihfzh888@sina.com (H.L.); 2 Yunnan Key Laboratory of Screening and Research on Anti-Pathogenic Plant Resources from Western Yunnan, Institute of Materia Medica, College of Pharmacy, Dali University, Dali 671000, China

**Keywords:** xanthone dimer, heterodimer, distribution, structure, pharmacology effect

## Abstract

Xanthone dimers, a distinctive class of natural metabolites renowned for their unique structures, are abundantly present in a diverse array of angiosperms, fungi, and lichens. These compounds not only exhibit remarkable diversity but also possess a broad spectrum of biological activities. In this comprehensive review spanning from 1966 to 2024, we synthesized the relevant literature to delve into the natural occurrence, biological potency, molecular structure and chemical diversity of xanthone dimers. The aim of this review is to serve as an insightful reference point for future scientific inquiries into xanthone dimers and their potential applications.

## 1. Introduction

In nature, organisms such as plants, fungi, and lichens are a rich treasure trove of natural products that have long been an important source for scientists to explore the diversity of new drug molecules, bioactive compounds, and chemical structures. Among them, xanthone dimers, as a class of natural compounds with a unique structure and a wide range of biological activities, have garnered considerable academic interest in recent years. With their complex molecular framework, diverse substitution modes, and significant pharmacological activity, these compounds have shown great potential in the fields of medicinal chemistry, natural product chemistry, and biomedicine.

Xanthones, a pivotal chemical constituent in the Clusiaceae family, constitute a diverse class of polyphenols with a ubiquitous distribution [1]. This class of compounds features two benzene rings fused to a pyrone ring. The structural versatility of xanthones is further augmented by the substitution of methoxy, hydroxy, and prenyl groups on the benzene rings [2,3]. The tricyclic framework enables xanthones to interact with various biomolecules, eliciting a wide array of biological activities, including antibacterial [4,5], anticancer [6,7], antioxidant [2,8,9], neuroprotective [8,9], hypoglycemic effects [10,11], and more.

Xanthone dimers, composed of two xanthone units, are commonly found in a variety of angiosperms, fungi, and lichens, particularly in medicinal plants native to tropical and subtropical regions. Plants like *Garcinia*, *Calophyllum*, *Hypericum*, *Mangifera*, and *Swertia* from the Clusiaceae family, along with certain fungal and lichen species, are known to be primary producers of xanthone dimers. The distribution characteristics of these natural products not only reflect biogeographical diversity but also serve as a valuable basis for future resource development and utilization. Notably, secalonic acids were first isolated in 1960 and exhibited intriguing biological activities [12]. As of 2024, researchers have since isolated 214 natural xanthone dimers from angiosperm species, along with various fungi and lichens. Based on the distinct monomers comprising their structures, xanthone dimers can be broadly classified into two groups: dimers and heterodimers. Dimers, commonly referred to as bis-xanthones, encompass 137 compounds consisting of two xanthone units. On the other hand, heterodimers consist of a xanthone unit paired with a non-xanthone unit, such as xanthonelignans and xanthone benzophenones [11]. There are a total of 77 identified heterodimers, with 20 of them being xanthone-derivative dimers.

In recent years, as research on xanthone dimers has deepened, their diverse biological activities have increasingly come to light. Xanthone dimers exhibit a wide array of biological effects, including anticancer [13,14,15,16], antibacterial [17,18,19,20], antioxidant [21,22] and neuroprotective [23,24] activities. In particular, their antitumor activity has become a focal point in the field of anticancer drug research and development due to their ability to modulate the cell cycle, induce apoptosis, inhibit angiogenesis, and other mechanisms. For example, griffipavixanthone (GPX, **28**) has garnered significant attention from researchers worldwide for its potent cytotoxicity against various human cancer cell lines, including lung, esophageal, and breast cancer cells, while showing minimal toxic side effects on normal cells. Furthermore, GPX (**28**) significantly inhibits tumor migration, invasion, and proliferation, both in vitro and in vivo [14,15,25]. Additionally, the notable effects of xanthone dimers in antioxidants and anti-inflammatories also provide new perspectives for the therapeutic strategies for addressing chronic ailments, encompassing cardiovascular diseases and the sequelae of diabetes mellitus. 

Given the immense potential of xanthone dimers in the field of medicine, the research advancements surrounding these compounds not only broaden our comprehension of chemical diversity in nature but also pave a novel path for new drug development. Previous comprehensive reviews in this area have provided valuable summaries of the research progress to date [26,27]. However, the aim of this article is to build upon these exiting foundations by offering an updated review of the research progress in this field over recent years, encompassing their distribution, structural characteristics, and biological activities. In particular, this article strives to present novel insights and inspiration for future in-depth investigations that may have been overlooked or under-explored in previous reviews.

**Figure 1:**
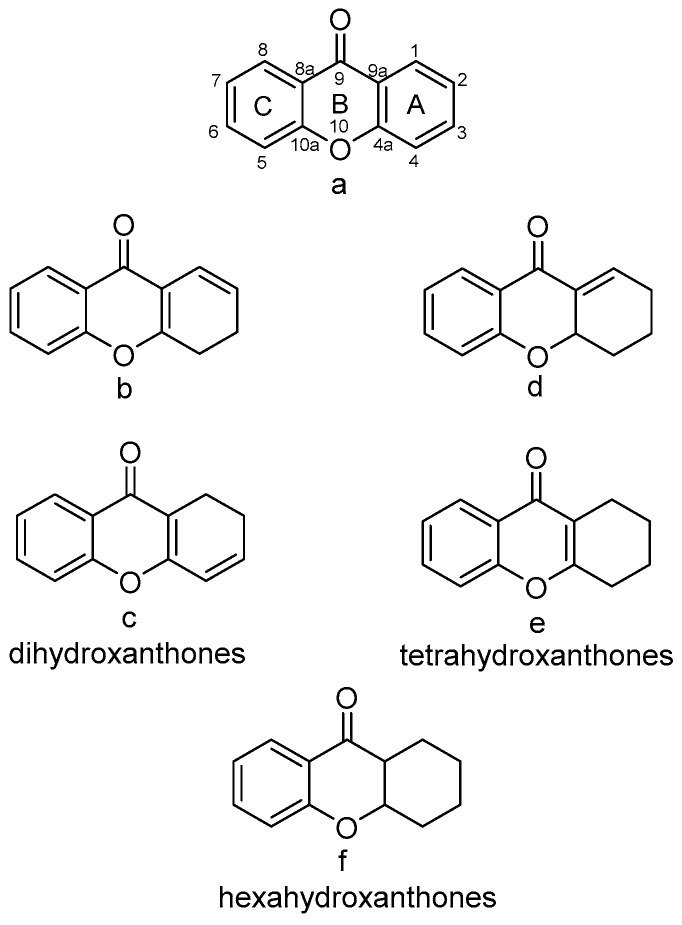
Xanthone subcategories (“a” represents xanthone, “b/c” represent dihydroxanthone, “d/e” represent tetrahydroxanthone, and “f” represents hexahydroxanthone).

As the numbering in the literature is not uniform, we will use Figure 1 for numbering in this paper. This approach aligns with the 2004 IUPAC Interim Recommendation for the parent compound 9*H*-xanthen-9-one [28].

## 2. Distribution

Xanthone dimers, a naturally occurring secondary metabolite, are ubiquitous in diverse plants and fungi belonging to various families. As presented in Table 1, the distribution of these compounds in plant and fungal species is detailed. This paper compiles a total of 214 naturally occurring xanthone dimers. More specifically, the current review uncovers 54 xanthone dimers originating from angiosperms, representing 25.23% of the total. Additionally, there are 15 xanthone dimers derived from lichens, constituting 7.01%. Notably, fungi are the primary source of xanthone dimers, with 145 compounds originating from fungal species, accounting for 67.76%, as detailed in Table 2.

## 3. Structural Characteristics and Classification

According to their structural characteristics, the xanthone family can be divided into the following categories [28]: firstly, they are divided into monomers and dimers/heterodimers. Then, according to the degree of oxidation of the C-ring of xanthone, they can be divided into four subcategories: full aromatic, dihydro, tetrahydro, and hexahydro–xanthone derivatives (e.g., a, b/c, d/e, f; see Figure 1). 

Xanthone dimers can be categorized into two primary groups: dimers and heterodimers, depending on their constituent monomers [11]. 

### 3.1. Dimer

Dimers typically refer to bis-xanthones, which are composed entirely of two xanthone units. These bis-xanthones are formed through the linkage of two xanthone moieties, resulting in a dimeric structure that retains the characteristic features of both individual xanthone units.

#### 3.1.1. Xanthone–Xanthone Dimers (a–a)

**Figure 2:**
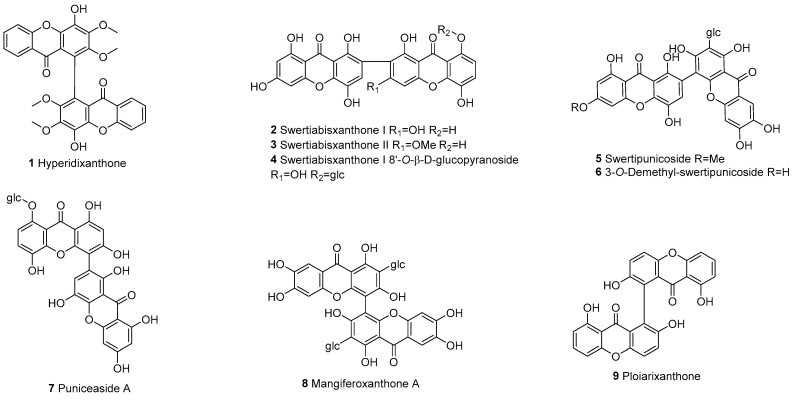

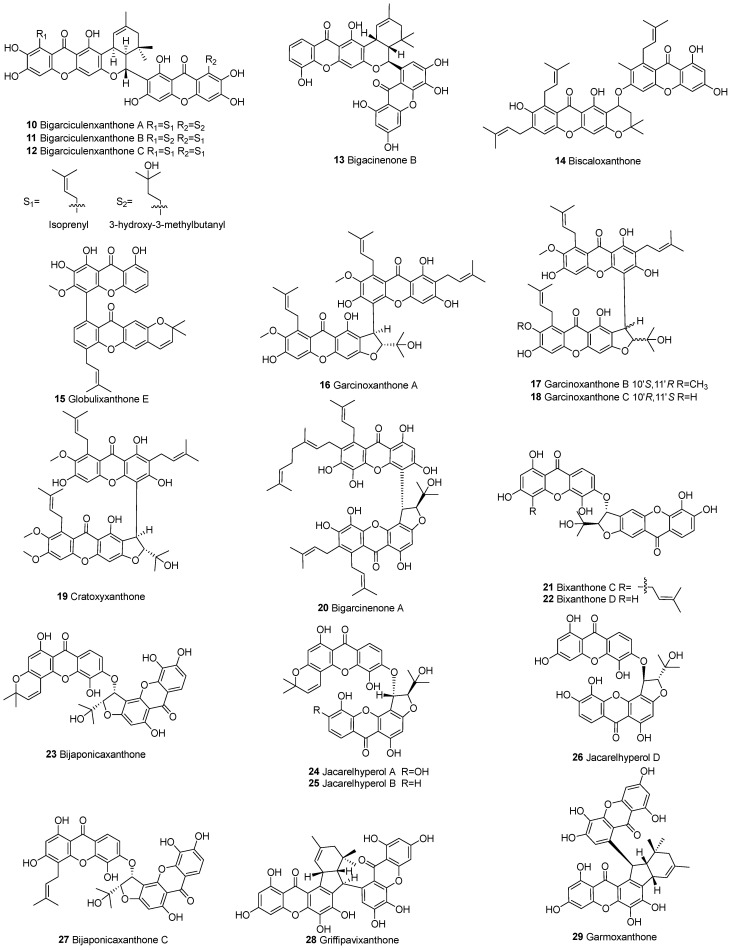

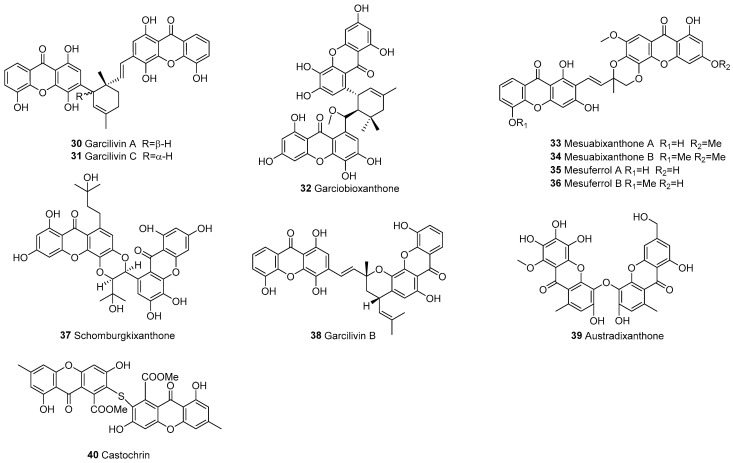
Xanthone–xanthone dimers (a–a).

Xanthone–xanthone of dimers formed by the polymerization of two intact xanthone (a), mainly derived from angiosperms, is represented by a total of 40 compounds, with 38 of them being derived from angiosperms (Figure 2). 

Xanthone dimers possess a wide range of linkage patterns, including aryl C–C bond linkages, aryl ether C–O–C linkages, aryl–O–alkyl linkages, and linkages formed by two isopentenyl derivatives [27]. Among these, there are also some noteworthy unique linkages. For instance, bigarcinenone B (**13**) is the first xanthone dimer to connect two perylene ketone units via a terpene bridge, achieved through two isopentenyl cyclization reactions [37]. Griffipavixanthone (**28**) is a unique xanthone dimer where one xanthone is tethered to another through a tandem cyclization process involving an isopentenyl group [111]. Garcilivins A and C (**30**–**31**) are composed of two xanthones linked by a terpene bridge [47]. Schomburgkixanthone (**37**) [52] is a novel bixanthone in which the two xanthone units are connected by a diether linkage. There are also individual xanthone dimers linked by S atoms, such as castochrin (**40**). This diverse array of attachment modes contributes significantly to the rich structural diversity exhibited by xanthone dimers. 

#### 3.1.2. Xanthone–Tetrahydroxanthone Dimers (a–e)

**Figure 3:**
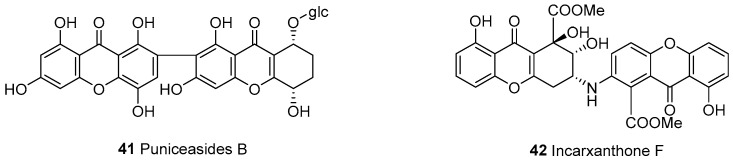
Xanthone–tetrahydroxanthone dimers (a–e).

In 2010, puniceaside B (**41**), a dimeric xanthone *O*-glycoside, was isolated from *Swertia punicea* [23]. The structure of **41** was characterized as 8-(*β*-D-glucopyranosyloxy)5,6,7,8-tetrahydro-1,3,5,1’,3’,5’,8’-heptahydroxy-[2,7’-bi-9*H*-xanthene]9,9’-dione. More recently, incarxanthone F (**42**) was isolated from the mangrove-derived endophytic fungus *Peniophora incarnata* Z4, which is linked by a C–N bond [55] (Figure 3).

#### 3.1.3. Dimeric Dihydroxanthones (b–b)

**Figure 4:**
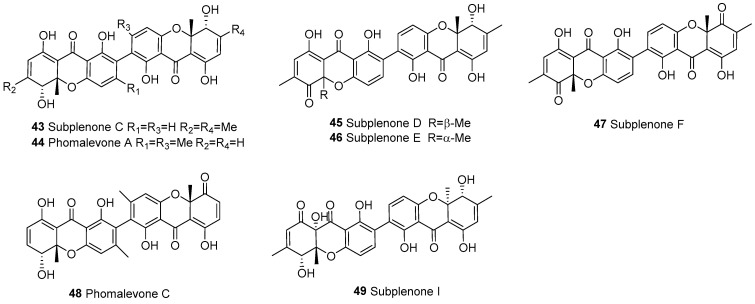
Dimeric dihydroxanthones (b–b).

Dihydroxanthones are relatively rare compounds, and according to current data, they are only derived from fungi. There are a total of seven dimers formed by the polymerization of dihydroxanthone, as shown in Figure 4. Subplenones C (**43**), D (**45**), E (**46**), F (**47**), and I (**49**) were isolated from *Subplenodomus* sp. CPCC 401465 [56]. Phomalevones A (**44**) and C (**48**) were isolated from a fungicolous Hawaiian isolate of *Phoma* sp.

#### 3.1.4. Dihydroxanthone–Tetrahydroxanthone Dimers (b–d or c–e)

**Figure 5:**
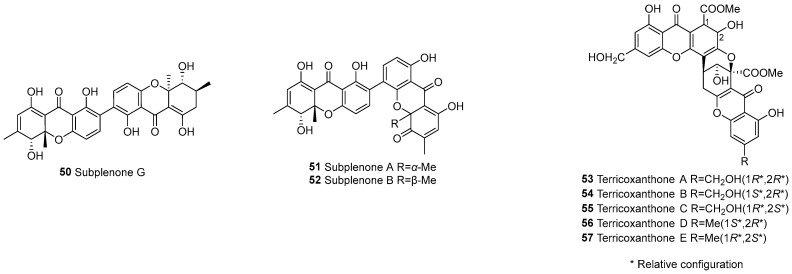
Dihydroxanthone–tetrahydroxanthone dimers (b–d or c–e).

The monomer types of the combination include b–d and c–e (Figure 5). In 2023, Cai et al. [56] isolated subplenones A (**51**), B (**52**), and G (**50**) from the endophytic fungus *Subplenodomus* sp. CPCC 401465, which resides within the Chinese medicinal plant *Gentiana straminea*. Terricoxanthones A–E (**53**–**57**) were isolated from the endophytic fungus *Neurospora terricola* HDF-Br-2 and were unprecedentedly dihydropyran-containing [58]. 

#### 3.1.5. Dimeric Tetrahydroxanthones (d–d or e–e)

**Figure 6:**
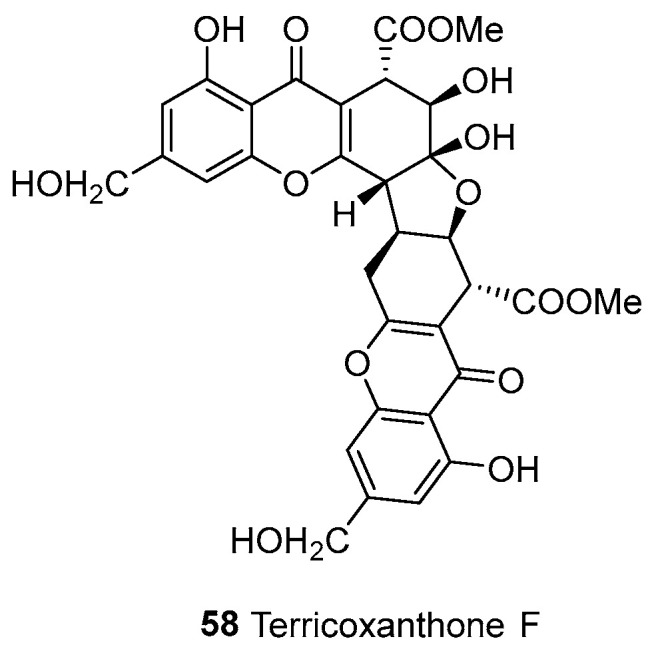
Dimeric tetrahydroxanthone (e–e).

**Figure 7:**
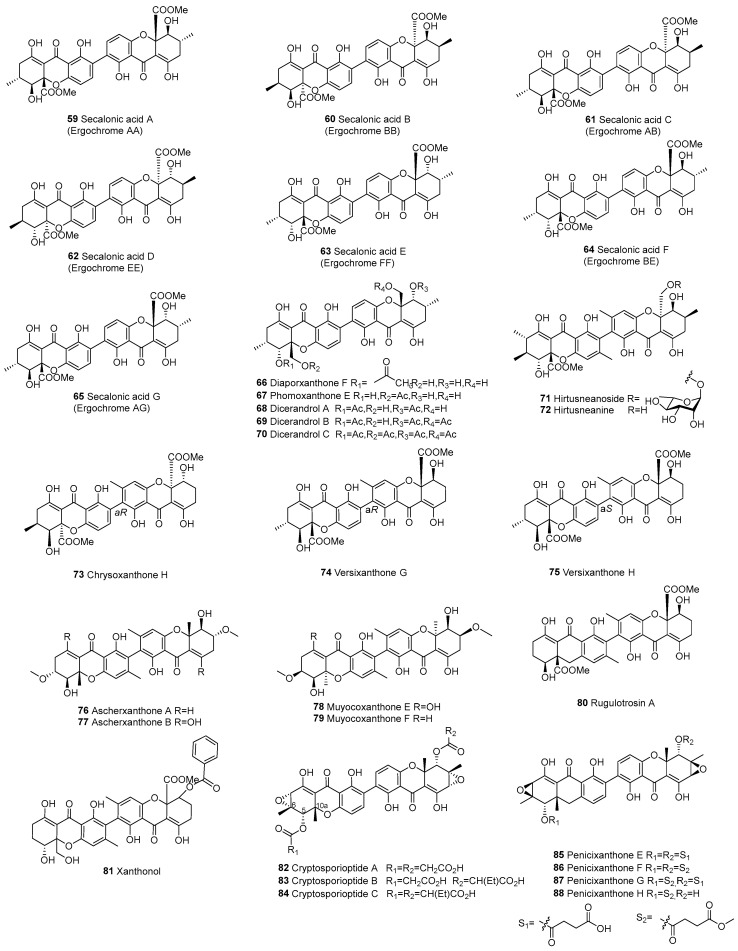

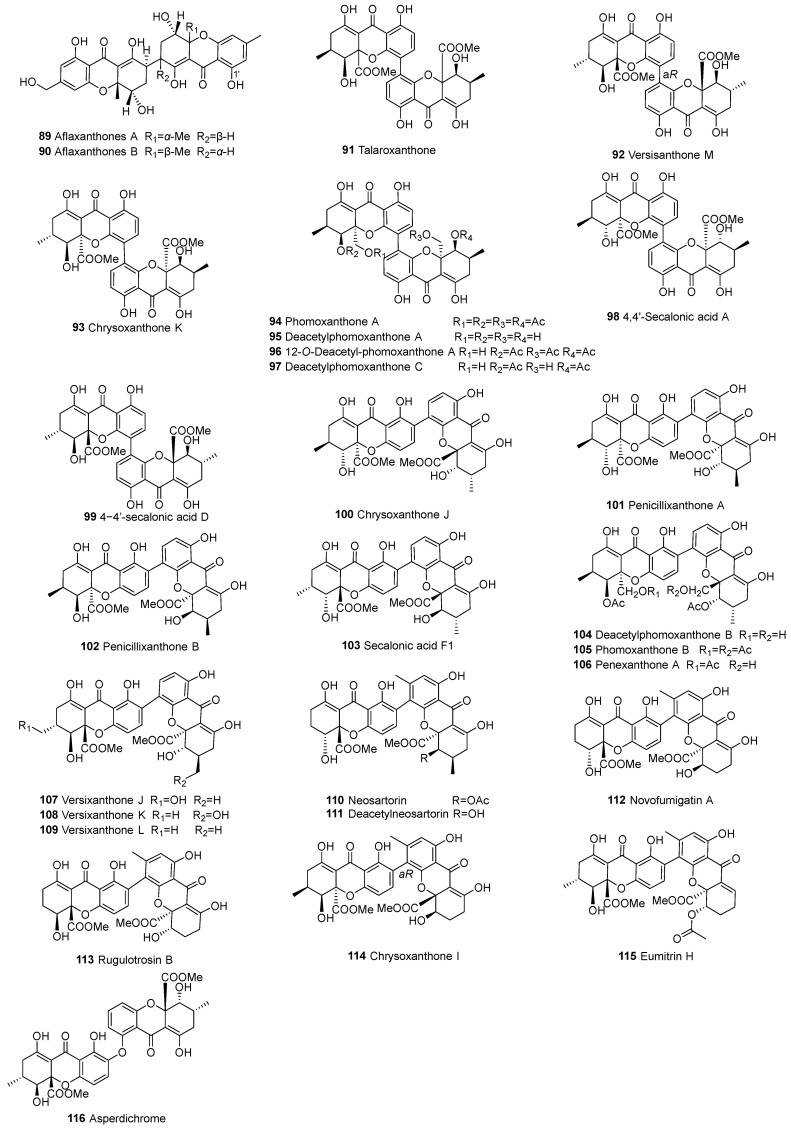
Dimeric tetrahydroxanthones (d–d).

Dimers formed by the polymerization of tetrahydroxanthone, composed of both parts of tetrahydroxanthone—and there are 59 of them—can be seen in Figure 6 and Figure 7. Tetrahydroxanthone dimers are primarily found in fungi and a few in lichens. Terricoxanthone F (**58**), a rare tetrahydrofuran-containing dimeric xanthone produced by the endophytic fungus *N. terricola* HDF-Br-2, had its physical and chemical properties, NMR spectra, and X-ray crystallographic data first described by Chen et al. in 2024 [58]. Asperdichrome (**116**), a tetrahydroperhydrone dimer linked by an ether bond, was isolated from a culture broth of *Aspergillus* sp. TPU1343 [62].

#### 3.1.6. Tetrahydroxanthone (d)–Hexahydroxanthone (f) Dimers

**Figure 8:**
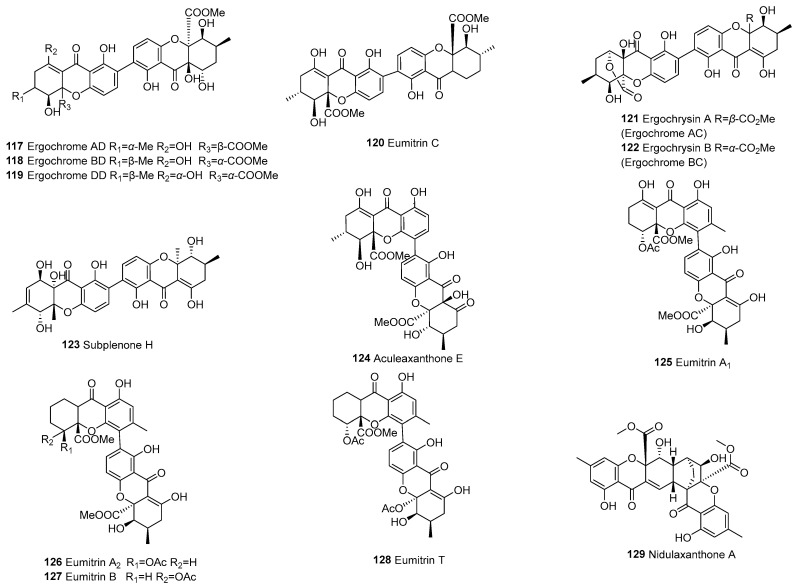
Tetrahydroxanthone (d)–hexahydroxanthone (f) dimers (d–f).

The dimer is formed by the polymerization of tetrahydroxanthone (d) and hexahydroxanthone (f), resulting in a total of 13, as shown in Figure 8. The primary linkage types in this dimer are C2–C2’ and C4–C2’. In 1973, eumitrin A_1_ (**125**), A_2_ (**126**), and B (**127**) were isolated from the lichen *Usnea bayleyi* (Stirt.) Zahlbr. More recently, in 2013, nidulaxanthone A (**129**), a xanthone dimer featuring a heptacyclic 6/6/6/6/6/6/6 ring system, was isolated from *Aspergillus* sp. F029. It is plausible that **129** arises through a [4+2] cycloaddition of its precursor nidulalin A [98]. 

#### 3.1.7. Dimeric Hexahydroxanthones (f–f)

**Figure 9:**
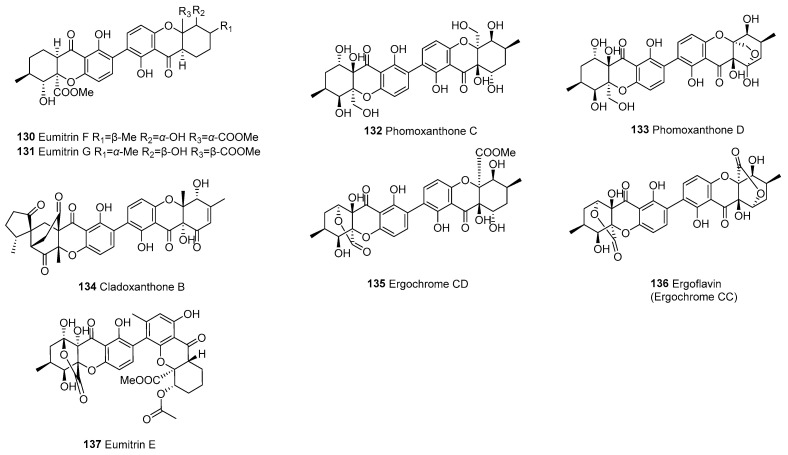
Dimeric hexahydroxanthones (f–f).

Eight dimers are formed by the polymerization of two hexahydroxanthones, as shown in Figure 9. In 2009, researchers isolated ergoflavin (**136**) from an endophytic fungus grown on the leaves of the Indian medicinal plant *Mimosops elengi* (bakul) [100]. Ergochrome CD (**135**) and ergoflavin (**136**) belong to a class of compounds called ergochromes, which are dimeric xanthenes linked in position 2. In 2022, cladoxanthone B (**134**), featuring a new spiro[cyclopentane-1,2’-[3,9a] ethanoxanthene]-2,4’,9’,11’(4a’*H*)-tetraone skeleton, was isolated from cultures of the ascomycete fungus *Cladosporium* sp. [99].

### 3.2. Heterodimers

Heterodimers are compounds that consist of two different monomers linked together. In the context of natural products chemistry, heterodimers often involve the combination of xanthones with other non-xanthone compounds or with other xanthone-related structures. 

#### 3.2.1. Xanthone–Flavone Heterodimers

**Figure 10:**
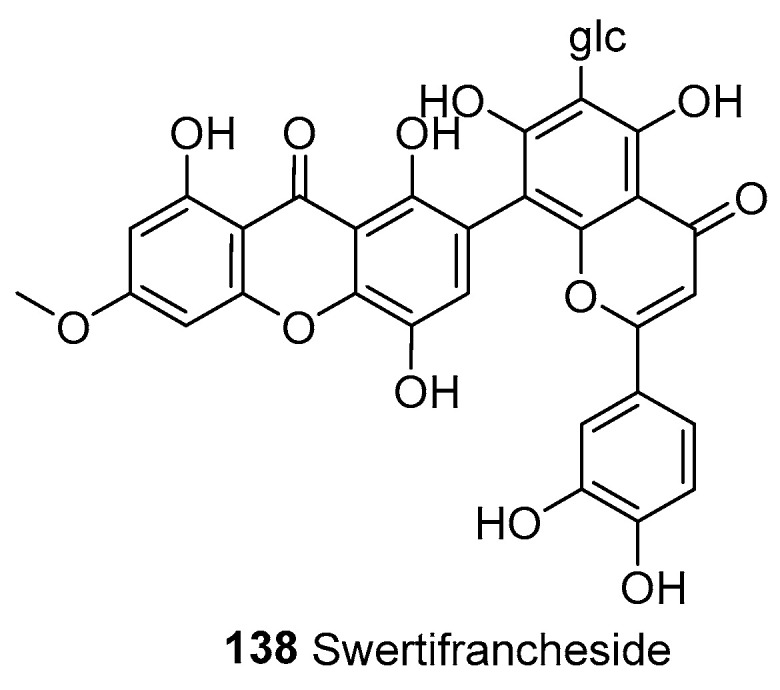
Xanthone–flavone heterodimer.

In 1994, swertifrancheside (**138**) was isolated from *Swertia franchetiana* and was the first identified xanthone–flavone C-glucoside [33]. Its structure was elucidated as 1,5,8-trihydroxy-3-methoxy-7-(5′,7′,3″,4″-tetrahydroxy-6′-C-β-d-glucopyranosyl-4′-oxy-8′-flavyl)-xanthone, as shown Figure 10. 

#### 3.2.2. Xanthonelignans

**Figure 11:**
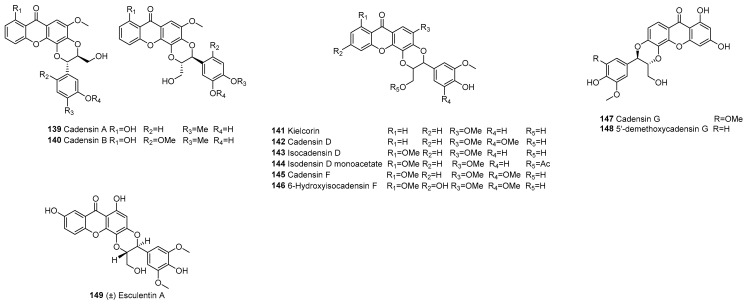
Xanthonelignan heterodimers.

In 1977, cadensins A (**139**), B (**140**), and kielcorin (**141**) were isolated respectively from *Caraipa aknsiflora* and *Kielmeyera coriacea*. The structure of (5*S*,6*S*)-6(or 5)-hydroxymethyl-5(or 6)-(4″-hydroxy-3″-methoxyphenyl)-2,3:3′,4′-(2′-methoxyxanthono)-l,4-dioxane was proposed for kielcorin by analysis of high resolution MS and PMR spectra [101]. In 1989, **142**–**147** were isolated from *Psorospermum febrifugum*. In 2014, (±) esculentin A (**149**) was isolated from *Garcina esculenta*, and it is the first xanthonolignoid from the genus *Garcinia* (Figure 11).

#### 3.2.3. Xanthone–Benzophenone Heterodimers

**Figure 12:**
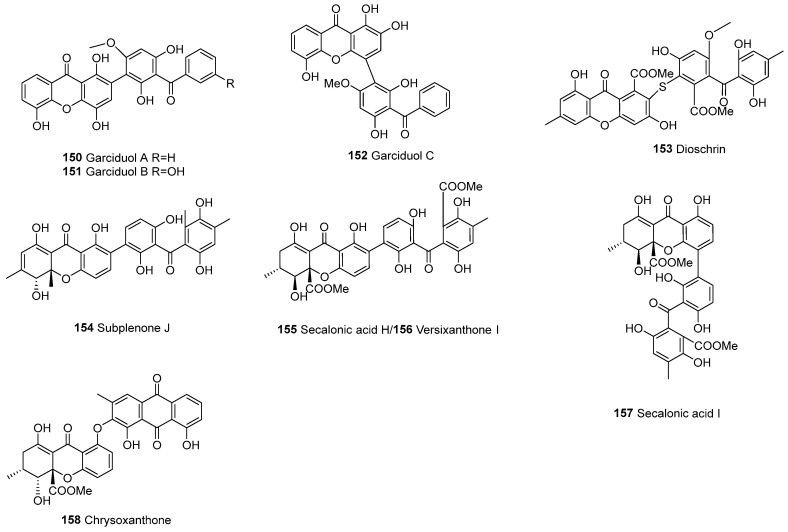
Xanthone–benzophenone heterodimers.

In 1996, garciduols A–C (**150**–**152**) were isolated from the roots of *Garcinia duicis* [103]. Dioschrin (**153**), linked by a thioether bond, was purified from an *Alternaria* sp. isolate obtained from a Hawaiian soil sample [54]. Versixanthone I (**156**) was the first tetrahydroxanthone–benzophenone heterodimer to be characterized and was isolated from *Aspergillus versicolor* HDN1009 [76] (Figure 12).

#### 3.2.4. Xanthone–Chromanone Heterodimers

**Figure 13:**
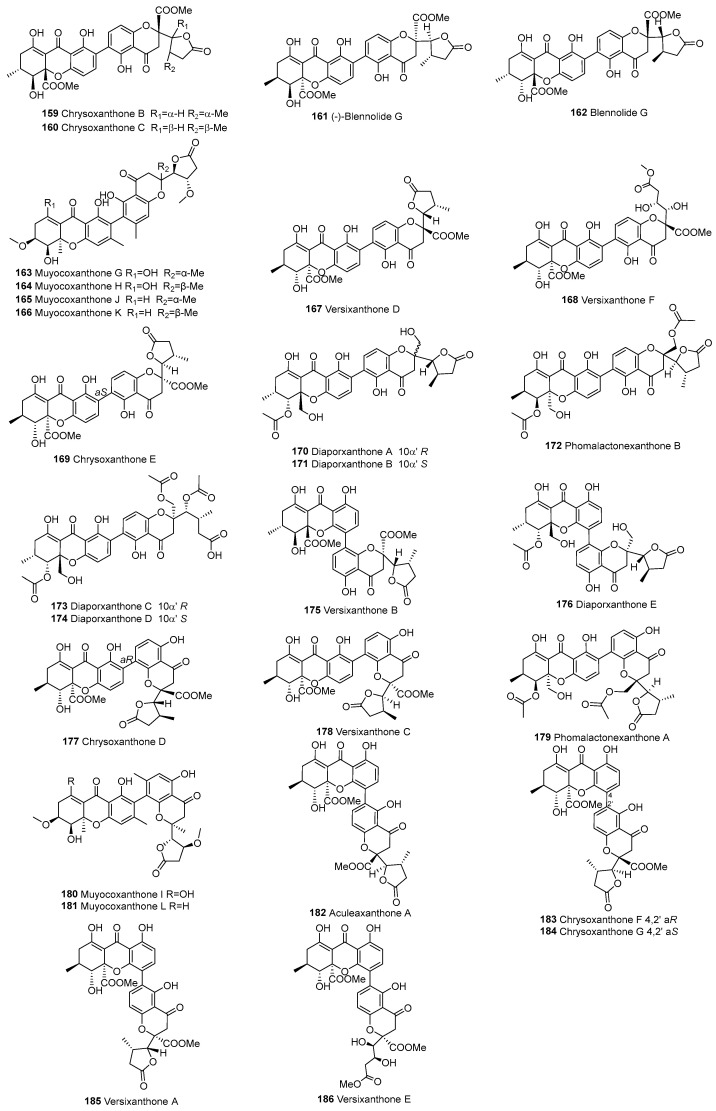
Tetrahydroxanthone (d/e)–chromanone heterodimers.

**Figure 14:**
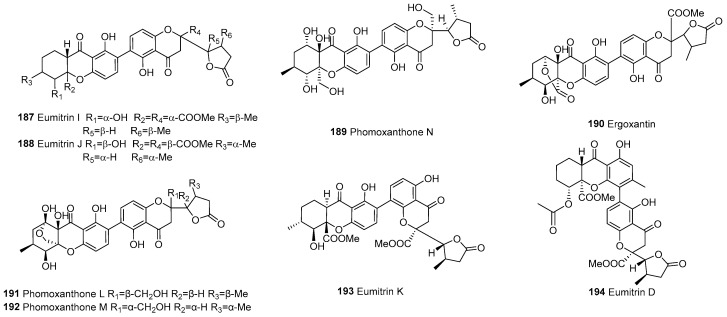
Hexahydroxanthone (f)–chromanone heterodimers.

In 2008, the heterodimer blennolide G (**162**) was isolated from *Blennoria* sp., an endophytic fungus from *Carpobrotus edulis*. The heterodimer **162**, composed of the monomeric blennolide A and the rearranged 11-dehydroxy derivative of blennolide E, extends the ergochrome family with an ergoxanthin type of skeleton [106]. So far, 36 xanthone–chromanone heterodimers have been published, categorized into two main groups (see Figure 13 and Figure 14).

#### 3.2.5. Dimeric Xanthone Derivatives (Chromanone–Chromanone Dimers)

**Figure 15:**
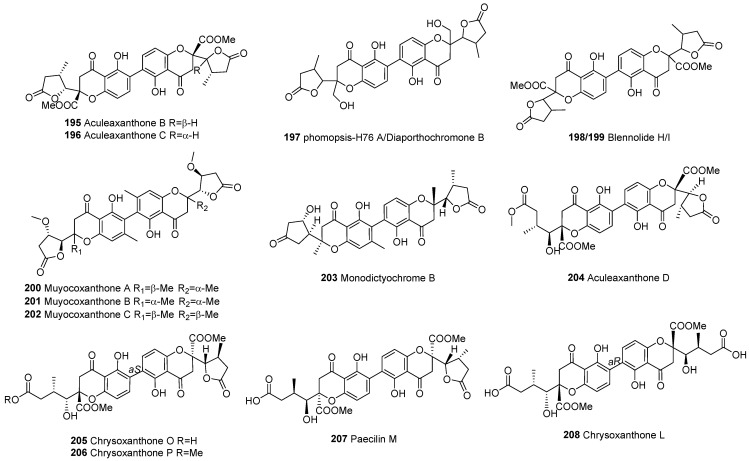

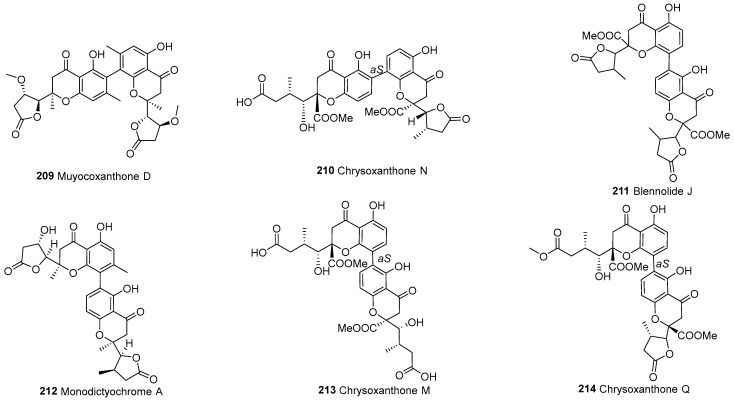
Dimeric xanthone derivatives.

In 2010, phomopsis-H76 A (**197**) was isolated from the mangrove endophytic fungus *Phomopsis* sp. (#zsu-H76) [109]. In subsequent years, such compounds have been reported, totaling 20 dimeric chromanones to date, as shown in Figure 15.

## 4. Pharmacology Effects

**Figure 16:**
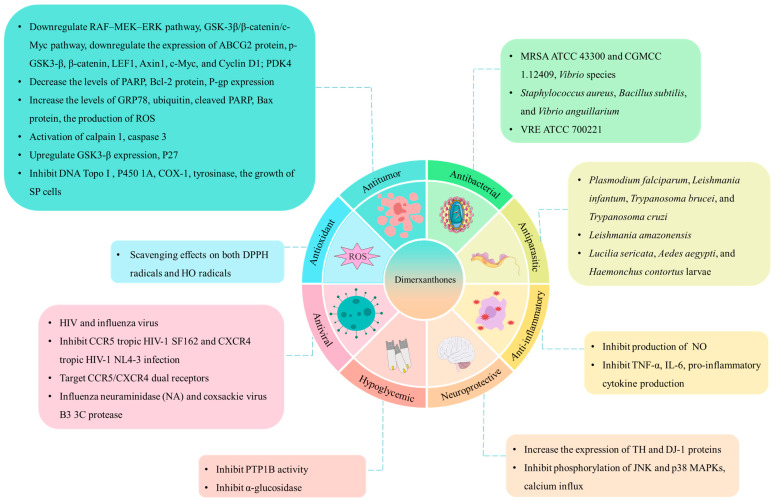
Main pharmacological activities of xanthone dimers and their corresponding activity mechanisms.

Xanthone dimers, a unique class of compounds, have attracted significant research attention for their remarkable biological activities across various fields. Their diverse bioactivities, such as anticancer, antibacterial, and anti-inflammatory properties, have sparked the interest of researchers and highlighted the potential medicinal and health applications of xanthone dimers. To gain a comprehensive and thorough understanding of these diverse bioactivities, we have compiled a comprehensive summary in Table 3. The main mechanism of drug activity of xanthone dimers is shown in Figure 16.

### 4.1. Antitumor Activity

According to the available literature, xanthone dimers exhibit inhibitory effects on the growth and reproduction of numerous tumor cell types, indicating significant clinical application potential.

**Figure 17:**
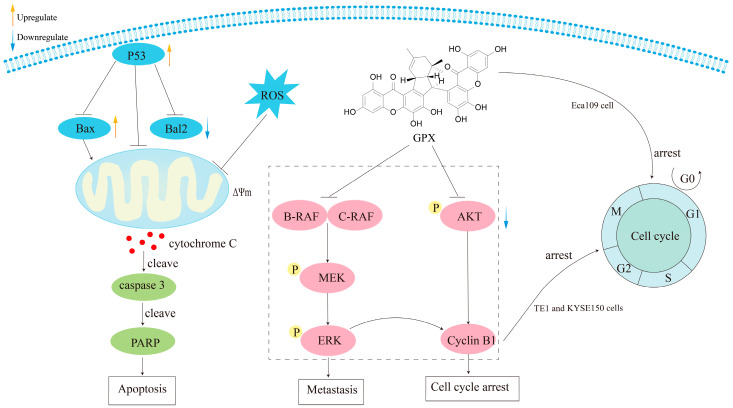
Mechanism of anticancer action of griffipavixanthone (GPX).

Notably, griffipavixanthone (GPX, **28**), a xanthone dimer derived from diverse *Garcinia* plant species, demonstrates potent antitumor properties in both in vitro and in vivo settings. Shi et al. [25] isolated GPX (**28**) from *G. oblongifolia* and discovered that it inhibits the proliferation of human non-small-cell lung cancer H520 cells in a dose- and time-dependent manner. Further mechanistic studies revealed that GPX triggers apoptosis via the mitochondrial apoptotic pathway, accompanied by the generation of reactive oxygen species (ROS). Ding et al. [15] isolated GPX (**28**) from *G. esculenta* and demonstrated its efficacy as an esophageal cancer cytostatic inhibitor of B-RAF and C-RAF. Various experimental assays showed that GPX inhibits cancer metastasis and proliferation, and intraperitoneal injection of GPX significantly reduced esophageal tumor metastasis and ERK protein levels in a lung metastasis model. Additionally, Ma et al. [14] found that GPX (**28**) exhibited lower toxicity towards normal breast cells, induced apoptosis in MCF-7 cells, and suppressed MCF-7 invasion and migration. Given these promising findings, GPX (**28**) holds potential as a therapeutic agent for lung, esophageal, and breast cancers (Figure 17).

Phomoxanthone analogs, belonging to the distinguished category of tetrahydroxanthone dimers derived from fungi, such as *Phomopsis* sp. and *Penicillium* sp., are regarded as structurally and biologically intriguing fungal xanthones [71]. Chen et al. [92] isolated phomoxanthone B (PXB, **105**) from the endophytic fungus *Phomopsis* sp. BCC By254, which inhibits the migration and invasion of human breast cancer cells MCF7, and has therapeutic potential for the treatment of estrogen receptor (ER)-positive breast cancer. Isaka et al. [87] conducted cytotoxicity studies on phomoxanthones A (**94**) and B (**105**) isolated from *Phomopsis* sp. BCC 1323, using the colorimetric method; it was found that both compounds were significantly cytotoxic to human breast cancer cells BC-1. The half-maximal inhibitory concentration (IC_50_) for the compounds was determined to be 0.51 and 1.70 μM, respectively. In addition, 12-*O*-deacetylphomoxanthone A (12-ODPXA, **96**), as a deacetylated derivative of phomoxanthone A (**94**), inhibits ovarian tumor growth and metastasis by downregulating PDK4, revealing the potential mechanisms of action of 12-ODPXA in ovarian cancer (OC) [123]. Furthermore, Ding et al. [71] employed the MTT assay to evaluate the cytotoxicity of the metabolites of *Phomopsis* sp. HNY29-2B and found that dicerandrols A and B (**68**–**69**), deacetylphomoxanthone B (**104**), and penexanthone A (**106**) exhibited cytotoxic activity (IC_50_ < 10 μM) against a broad range of cell lines, including MDA-MB-435 (human breast cancer), HCT-116 (human colon cancer), Calu-3 (human lung cancer), and Huh-7 (human hepatocellular carcinoma). In subsequent studies, Gao et al. [13] discovered that dicerandrol B (**69**) induces apoptosis in cervical cancer HeLa cells, highlighting its anticancer potential, specifically targeting cervical cancer through ER stress and mitochondrial apoptosis. Zhao et al. [124] proposed that penexanthone A (**106**) enhanced the sensitivity of CRC to CDDP and induced ferroptosis by targeting Nrf2 inhibition, indicating that PXA might serve as a novel anticancer drug in combination chemotherapy. Zhou et al. [122] revealed that dicerandrol C (**70**) inhibits proliferation and induces apoptosis in liver and cervical cancer cells, potentially via GSK3-β-mediated Wnt/β-catenin signaling. This finding provides profound insights into the underlying mechanisms responsible for the effective efficacy of dicerandrol C (**70**) in the context of hepatocellular and cervical cancer. Therefore, the phomoxanthone family as a whole holds immense promise in the realm of antitumor therapy.

DNA topoisomerase I (Topo I) is an important target for anticancer drug development [76]. In 2011, Ren et al. [119] first investigated the inhibitory activity of secalonic acid D (SAD, **62**) on Topo I. The results showed that it exhibited strong inhibitory activity against Topo I in a dose-dependent manner, and the minimum inhibitory concentration (MIC) was 0.4 μM. Distinct from the archetypal DNA Topo I inhibitor camptothecin (CPT), SAD inhibited the Topo I–DNA binding interaction without eliciting the formation of covalent Topo I–DNA complexes. Furthermore, versixanthones G (**74**), H (**75**), and K (**108**), isolated from the marine fungus *Aspergillus vericolor*, exhibited inhibitory activity against Topo I. Notably, versixanthone G (**74**) demonstrated a concentration-dependent effect. Mechanistic studies illuminated that versixanthone G functions by sequestering Topo I–DNA complexes, arresting the cell cycle at the G2/M phase, and triggering necrosis in cancer cells. These findings underscore its potential as a leading template for the development of novel Topo I inhibitors [76].

**Figure 18:**
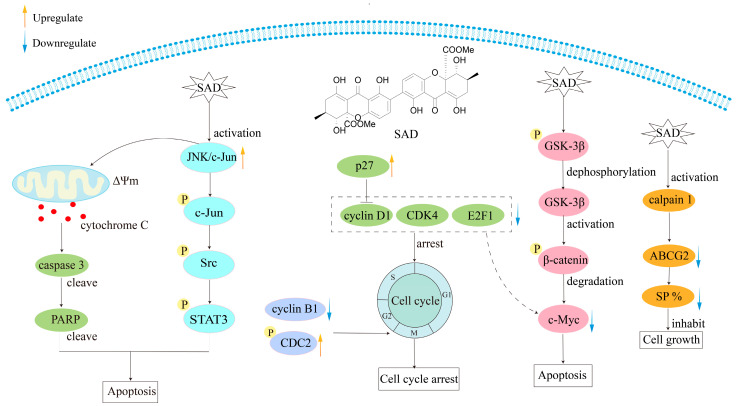
Mechanism of antitumor action of secalonic acid D (SAD).

Secalonic acid D (SAD, **62**), a prominent environmental toxin, isolated from *Penicillium oxalate*, a prevalent microbial contaminant in freshly harvested maize, has been documented to have acute toxic and teratogenic properties [125]. Due to the lack of studies on its antitumor activity, Zhang et al. [116] in 2009 isolated SAD from the secondary metabolite of the mangrove endophytic fungus No. ZSU44, which showed strong cytotoxicity against the human leukemia cell lines HL60 and K562 cells, with IC_50_ values of 0.38 and 0.43 μmol/L, respectively. Employing Annexin V-FITC/PI assay and protein Western blot (WB) analysis, the results showed that it triggers apoptosis and arrests the cell cycle at the G1 phase in leukemia cells through the GSK-3*β*/*β*-catenin/c-Myc signaling pathway. In 2013, Hu et al. [118] further demonstrated SAD’s robust cytotoxic activity against side populations (SPs) by inducing the degradation of ATP-binding cassette transporter subfamily G member 2 (ABCG2) by activating calpain 1. More recently, Zhang et al. [119] identified SAD as highly cytotoxic to three pairs of multidrug-resistant (MDR) cells and their parental sensitive counterparts, including S1-MI-80 and S1, H460/MX20 and H460, and MCF-7/ADR and MCF-7 cells. SAD induces cancer cell death through the c-Jun/Src/STAT3 signaling axis, inhibiting proteasome-dependent degradation of c-Jun in sensitive cells and overcoming ABCG2-mediated MDR (Figure 18).

Beyond the aforementioned xanthone dimers, some heterodimers have exhibited notable anticancer activity as well. Wu et al. [16] first isolated six unique xanthone–chromanone dimers, versixanthones A-F (**185**, **175**, **178**, **167**, **186**, **168**), from cultures of the mangrove-derived fungus *A. versicolor* HDN1009. These compounds contain tetrahydroxanthone and 2,2-disubstituted chromanone monomers linked in different forms. The cytotoxicity of the six compounds was assessed using the MTT assay. Versixanthones A-F were found to be potent against various cancer cell lines, including human promyelocytic leukemia HL-60, chronic myeloid leukemia K562, non-small-cell lung carcinoma A549 and H1975, gastric carcinoma 803, embryonic kidney HEK293, ovarian carcinoma HO8910, and colon carcinoma HCT-116. All of these compounds demonstrated cytotoxicity, with the most potent IC_50_ value being 0.7 μM. Moreover, Pontius et al. [110] isolated two dimeric chromanone compounds, monodictyochromes A (**212**) and B (**203**), from the fungus *Monodictys putredinis*. Their study revealed that both compounds inhibited cytochrome P450 1A activity with IC_50_ values of 5.3 and 7.5 μM, respectively.

### 4.2. Antibacterial Activity

Wang et al. [20] made a noteworthy discovery, revealing that garmoxanthone (**29**), derived from the pericarp of *G. mangostana*, exhibited potent inhibitory activity against methicillin-resistant *Staphylococcus aureus* (MRSA) strains ATCC 43300 and CGMCC 1.12409, with an MIC value of 3.9 μg/mL. Furthermore, it displayed a moderate inhibitory effect on *Vibrio* species, thus validating the potential of *G. mangostana* as a therapeutic agent against infections. In a parallel study, Augustin et al. [39] employed an agar diffusion assay to demonstrate the efficacy of globulixanthone E (**15**), isolated from the root bark of *Symphonia globulifera*, against Gram-positive bacteria, including *S. aureus*, *Bacillus subtilis*, and *Vibrio anguillarium*. The findings indicated that globulixanthone E (**15**) exhibited antimicrobial effects comparable to streptomycin, suggesting its potential as a natural antimicrobial agent.

Cai et al. [56] isolated subplenones A–J from the endophytic fungus *Subplenodomus* sp. CPCC 401465. Notably, subplenones A (**51**), E (**46**), and G (**50**) displayed particularly robust efficacy against MRSA ATCC 700698 and vancomycin-resistant *Enterococcus faecium* (VRE) ATCC 700221, with MIC values ranging from 0.25 μg/mL to 0.5–1.0 μg/mL, respectively.

*Aspergillus* species fungi, ubiquitous in diverse natural habitats, have garnered attention for their remarkable antibacterial properties. Wu et al. [19] isolated secalonic acid D (**62**) from *Aspergillus aculeatinus* WHUF0198, which displayed antibacterial activity against a broad spectrum of bacterial, including Gram-negative (*Helicobacter pylori* G27, *H. pylori* 26695, *H. pylori* 129) and Gram-positive bacteria (MRSA USA300 and *B. subtilis* 168), as well as multidrug-resistant strains (*H. pylori* 159), with MIC values ranging from 1.0 to 4.0 μg/mL. Zang et al. [18] isolated two new heterodimeric tetrahydroxanthone compounds, aflaxanthones A and B (**89**–**90**), from the mangrove-derived endophytic fungus *Aspergillus flavus* QQYZ. These compounds exhibited promising antifungal and antibacterial activities against *Candida albicans* and four agricultural plant pathogenic fungi (*Fusarium oxysporum*, *Penicillium italicum*, *Collettrichum musae*, and *Colletotrichum gloeosporioides*), with MIC values ranging from 3.13 to 25 μM. Notably, aflaxanthone A (**89**) also showed moderate antibacterial activity against MRSA and *B. subtilis*. Xu et al. [17] uncovered penicillixanthone A (**101**) in the marine-derived fungus *Aspergillus brunneoviolaceus* MF180246, which effectively inhibited the growth of *S. aureus* at an MIC of 6.25 μg/mL. These findings highlight that xanthone dimers in *Aspergillus* species possess good antibacterial potential and are promising lead compounds for the development of antibacterial drugs.

Moreover, phomoxanthone A (**94**) isolated from the endophytic fungus *Phomopsis* sp. BCC 1323 exhibited strong inhibitory activity against *Mycobacterium tuberculosis* (H37Ra strain) [87]. Rugulotrosins A (**80**) and B (**113**), extracted from *Penicillium* sp., demonstrated significant antibacterial efficacy against *B. subtilis*. Rugulotrosin A (**80**) also showed strong antibacterial capability against *Enterococcus faecalis* and *Bacillus cereus* [80]. Schüffler et al. [105] isolated chrysoxanthone (**158**) from the ascomycete IBWF11-95A, displaying antibacterial activity against various bacterial species. The MIC values ranged from 2.5 to 20 μg/mL, with *Arthrobacter citreus* being the most sensitive. It inhibited the growth of certain fungal species.

Lichen, an invaluable natural resource, has emerged as a rich source of xanthone dimers. For instance, hirtusneanoside (**71**), isolated from *Usnea hirta*, effectively inhibits the growth of Gram-positive bacteria, including *S. aureus* and *B. subtilis* [75]. Furthermore, Nguyen et al. [95] succeeded in obtaining eumitrins F–H (**130**, **131**, **115**) from the dichloromethane extract of *Usnea baileyi*, which exhibited moderate yet promising antibacterial characteristics. These findings underscore the diverse and potent antibacterial potential of xanthone-derived compounds sourced from lichen.

### 4.3. Antioxidant Activity

In 2008, Zhong et al. [22] first isolated bigarcinenone A (**20**) from the bark of *Garcinia xanthochymus*. This compound showcased remarkable antioxidant activity in the 1,1-diphenyl-2-picrylhydrazyl (DPPH) radical scavenging assay, with an IC_50_ value of 9.2 μM, surpassing the efficacy of the established butylated hydroxytoluene (BHT) twofold; BHT exhibited an IC_50_ of 20 μM. Notably, the study further demonstrated that the incorporation of hydroxy or catechol groups in related molecules further bolstered the radical scavenging capabilities. Building upon this, Chen et al. [37] isolated bigarcinenone B (**13**) from the bark of the same plant species. The researchers evaluated its antioxidant activity using both the DPPH radical scavenging method and the luminol-H_2_O_2_-CoII-EDTA chemiluminescence system. The findings revealed that the xanthone dimer possessed significant scavenging effects on both DPPH radicals and HO radicals, with IC_50_ values of 20.14 and 2.85 μM, respectively.

Moreover, Merza et al. [21] isolated griffipavixanthone (**28**) from the twigs of *Garcinia virgata* and studied its antioxidant activity. They found that it possessed high radical scavenging ability, with a median effect concentration (EC_50_) value as low as 11.5 μg/100 mL, outperforming the reference compounds butylated hydroxyanisole (BHA) and α-tocopherol. This underscores the significant antioxidant potential harbored within the *Garcinia* genus.

### 4.4. Anti-Inflammatory Activity

In 2016, Liu et al. [40] isolated three xanthone dimers, named garcinoxanthones A–C (**16**–**18**), from the pericarp of *Garcinia mangostana* collected in Thailand. The results of nitric oxide (NO) inhibition activity testing using lipopolysaccharide (LPS)-stimulated RAW264.7 showed that garcinoxanthones B and C (**17**–**18**) significantly inhibited NO production. Their IC_50_ values were 11.3 ± 1.7 and 18.0 ± 1.8 μM, respectively, which were comparable to the positive control drug indomethacin (IC_50_ of 3.9 ± 0.3 μM). Additionally, they found that garcinoxanthone B (**17**) could inhibit the expression of inducible NO synthase in a dose-dependent manner. These findings not only revealed the presence of rare xanthone dimers in *G. mangostana* but also demonstrated the inhibitory effect of these compounds on NO production in LPS-stimulated mouse macrophages.

### 4.5. Neuroprotective Effects

In 1989, swertiabisxanthone I (**2**) was first isolated from *Swertia macrosperma* [30]. A decade-and-a-half later, Hostettmann et al. [32] discovered its glycoside derivative within *Gentianella amarella* ssp. acuta, swertiabisxanthone I 8’-*O*-*β*-D-glucopyranoside (**4**). Subsequently, in 2010, Du et al. [23] isolated puniceaside B (**41**), swertiabisxanthone I 8’-*O*-*β*-D-glucopyranoside (**4**), and 3-*O*-demethylswertipunicoside (**6**) from the whole plant of *S. punicea*. Utilizing the MTT assay, the results demonstrated that puniceaside B (**41**) exhibited robust neuroprotective capabilities against H_2_O_2_-induced damage in rat pheochromocytoma cells (PC12). Furthermore, in September of the same year, Zhang et al. [24] further confirmed through MTT cell viability assays and acridine orange/ethidium bromide (AO/EB) apoptosis assays that 3-*O*-demethylswertipunicoside (**6**) exerts its potential neuroprotective effects by upregulating the expression of tyrosine hydroxylase (TH) and DJ-1 proteins. These cumulative discoveries underscore the neuroprotective potential of compounds derived from *Swertia* species, hinting at their therapeutic potential in addressing neurological disorders.

Secalonic acid A (SAA, **59**), a naturally occurring compound derived from marine fungi, has a protective effect against colchicine-induced apoptosis in rat cortical neurons. In 2011, Zhai et al. [113] examined the protective effect of SAA on 1 mM colchicine-treated cortical neurons using Hoechst 33258, LDH release, and flow cytometry. The results revealed that SAA of 3 and 10 mM significantly inhibited colchicine-induced apoptosis in cortical neurons. This protective mechanism of SAA likely involves the inhibition of c-Jun N-terminal kinase (JNK) and p38 mitogen-activated protein kinase (MAPK) phosphorylation, calcium influx, and calpain I activation, thereby counteracting the cytotoxic effects of colchicine on rat cortical neurons. In 2013, Zhai et al. [114] further demonstrated the protective effect of SAA in a mouse model of Parkinson’s disease. SAA protects against 1-methyl-4-phenyl-1,2,3,6-tetrahydropyridine (MPTP)-induced dopaminergic neuronal death and attenuates 1-methyl-4-phenylpyridinium (MPP^+^)-induced cytotoxicity in nigrostriatal neurons and human neuroblastoma SH-SY5Y cells. During MPP^+^-mediated apoptosis, SAA was found to inhibit JNK and p38 MAPK, downregulate Bax expression, and suppress calpain I activation. These findings suggest that SAA may rescue MPP^+^-induced dopaminergic neuronal death via modulation of the mitochondrial apoptotic pathway.

### 4.6. Hypoglycemic Effects

Protein tyrosine phosphatase 1B (PTP1B) plays a crucial role in negatively regulating insulin and leptin signaling pathways, making PTP1B inhibitors potential novel therapeutics for type 2 diabetes and obesity.

In 2016, Yamazaki et al. [62] made a groundbreaking discovery by isolating asperdichrome (**116**) from the fermentation culture of *Aspergillus* sp. TPU1343. Their meticulous investigation, which involved quantifying the hydrolysis rate of p-nitrophenyl phosphate (pNPP) as a PTP1B substrate, revealed that both asperdichrome (**116**) and the heterodimer secalonic acid F (**64**) potently inhibited PTP1B activity, exhibiting IC_50_ values of 6.0 and 9.6 μM, respectively. In contrast, the homodimer SAD (**62**) showed a lesser degree of inhibition effect, achieving 40% inhibition at 15.7 μM. This indicates that the combination of asperdichrome (**116**) and secalonic acid F (**64**) in a heterodimeric structure seems to be more effective in inhibiting activity than the homodimeric structure of SAD (**62**). It is worth noting that this study represents the first investigation into the PTP1B inhibitory properties of tetrahydroxanthone compounds. In 2017, Rotinsulu et al. [60] identified a new 2, 4′-linked tetrahydroxanthone dimer, secalonic acid F1 (**103**), from the same fungus *Aspergillus* sp. TPU1343. Through enzymatic activity assays, researchers confirmed that this compound effectively inhibited PTP1B activity, with an IC_50_ value of 5.9 μM, similar to the positive control, oleanolic acid (IC_50_ = 1.1 μM). This discovery highlights the potential of tetrahydroxanthone dimers as PTP1B inhibitors, offering promising lead molecules for developing therapeutic agents to address type 2 diabetes and obesity. In 2020, Lien et al. [52] isolated schomburgkixanthone (**37**) and GPX (**28**) from the branches of *Garcinia schomburgkiana*. They evaluated the in vitro inhibitory activity of these two compounds against rat intestinal α-glucosidase. Notably, schomburgkixanthone (**37**) had the most significant inhibitory effect on maltase and sucrase, with IC_50_ values of 0.79 and 1.81 μM, respectively. In contrast, GPX (**28**) displayed stronger inhibition against sucrase, with an IC_50_ value of 4.58 μM. These findings offer valuable clues for the development of new hypoglycemic therapeutic agents.

### 4.7. Antiviral Activity

Studies have shown that xanthone dimers exhibit significant antiviral activity, mainly against the human immunodeficiency virus (HIV) and influenza virus. For instance, *S. franchetiana*-derived swertifrancheside (**138**) has exhibited inhibitory activity in HIV-1 reverse transcriptase, achieving a staggering 99.8% inhibition at 200 μg/mL, with swertipunicoside (**5**) boasting an ED_50_ as low as 3.0 μg/mL [33]. Furthermore, marine-sourced penicillixanthone A (**101**), isolated from the jellyfish-derived fungus *Aspergillus fumigata*, was investigated using molecular docking techniques to explore its interaction with CCR5/CXCR4 receptors. The outcomes revealed that penicillixanthone A (**101**) was able to inhibit CCR5 tropic HIV-1 SF162 and CXCR4 tropic HIV-1 NL4-3 infection, exhibiting strong anti-HIV-1 activity with IC_50_ values of 0.36 μM and 0.26 μM, respectively. As a compound capable of simultaneously targeting CCR5/CXCR4 dual receptors, penicillixanthone A (**101**) presents a novel and promising candidate for anti-HIV drug development [91].

Abdel-Mageed et al. [34] also made a significant contribution by extracting mangiferoxanthone A (**8**) from the *n*-butanol fraction of the stem bark of *Mangifera indica*. An evaluation of its antiviral properties revealed moderate inhibitory effects against influenza neuraminidase (NA) and coxsackie virus B3 3C protease. Specifically, mangiferoxanthone A (**8**) demonstrated a 55.8% inhibition rate against influenza NA and a 46.1% inhibition rate against coxsackie virus B3 3C protease at a concentration of 100 μM, indicating its potential as an antiviral agent against these viral targets.

### 4.8. Antiparasitic Activity

Antiparasitic drugs are primarily categorized into distinct groups: anthelmintics, antiprotozoals, and insecticides. Researchers have isolated garcilivins A (**30**) and C (**31**) from the bark of *G. livingstonei*, and these two compounds have shown antiparasitic activity against *Plasmodium falciparum*, *Leishmania infantum*, *Trypanosoma brucei*, and *Trypanosoma cruzi* [47]. Additionally, phomoxanthones A (**94**) and B (**105**), isolated from the endophytic fungus *Phomopsis* sp. BCC 1323, have demonstrated significant inhibitory activity against *P*. *falciparum* [87]. Notably, phomoxanthone A (**94**), isolated from the plant endophytic fungus *Paecilomyces* sp. EJC01.1, effectively inhibits the promastigotes of *Leishmania amazonensis* (IC_50_ = 16.38 ± 1.079 μg/mL) and *T*. *cruzi* (IC_50_ = 28.61 ± 1.071 μg/mL) [74]. Furthermore, Ondeyka et al. isolated xanthonol (**81**), a novel xanthone dimer, from the fermentation broth of a non-sporulating fungus in 2006. Experimental results showed that it exhibits insecticidal and repellent activity against *Lucilia sericata*, *Aedes aegypti*, and *Haemonchus contortus* larvae, with LD_90_ values of 33, 8, and 50 μg/mL, respectively [81]. In a nutshell, these findings underscore the promising potential of xanthone dimers in the prevention, eradication, and elimination of parasitic infections.

### 4.9. Other Activities

In addition to the aforementioned biological activities, xanthone dimers also possess other valuable properties. For example, Zhu et al. [42] isolated GPX (**28**) from the ethyl acetate extract fraction of an 80% (*v*/*v*) ethanol extract of *Garcinia esculenta*, which exhibited strong xanthine oxidase (XO) inhibitory activity with an IC_50_ value of 6.3 μM. Moreover, GPX (**28**) is considered the first xanthone dimer compound to demonstrate strong XO inhibitory activity in vitro, and this inhibition is concentration dependent.

Zhang et al. discovered that bxanthones C (**21**) and D (**22**), which were isolated from *Auricium aurantium*, are active ingredients in traditional Chinese medicine used for treating various liver diseases. They also found that the acetone component of the plant has been utilized in the treatment of liver fibrosis [27]. Additionally, jacarelhyperols A (**24**) and B (**25**) were obtained from the chloroform extract of the methanol extract of *Hypericum japonicum*, and in vivo experiments confirmed their significant ability to inhibit platelet-activating factor (PAF) [44].

Furthermore, phomoxanthones D (**133**), L (**191**), M (**192**), and N (**189**) isolated from the co-culture of *Phomopsis asparagi* DHS-48 and *Phomopsis* sp. DHS-11 have demonstrated modest immunosuppressive activity against ConA-induced (T-cell) and LPS-induced (B-cell) mouse spleen lymphocyte proliferation [89]. These discoveries underscore the diverse and promising applications of dimerxanthones in various therapeutic and pharmacological avenues.

## 5. Conclusions and Prospects

By conducting a thorough review of current literature, we have gained a deep understanding of xanthone dimers—a group of natural compounds known for their unique chemical compositions, found in a wide range of angiosperms, fungi, and lichens. The wide variety of these sources has not only expanded the collection of natural products but also provided abundant resources for further exploration into their biosynthetic processes. Notably, recent studies in molecular biology and ecology have illuminated the intricate interplay between specific ecological niches and the distribution as well as the diversity of xanthone dimers, thereby laying a solid scientific foundation for future endeavors in resource development and conservation.

The literature review underscores the remarkable structural complexity and diversity of xanthone dimers, featuring a vast assortment of dimeric skeletons with differing linkage patterns, a myriad of substitution profiles, and intricate stereochemical variations. These unique structural attributes not only dictate their exceptional physicochemical properties but also underpin their diverse biological activities. As modern spectroscopic analysis techniques, including NMR and MS, continue to evolve, an ever-growing number of xanthone dimer structures are being precisely elucidated, furnishing robust data that underpin structure–activity relationship studies.

Xanthone dimers have garnered considerable attention owing to their panoply of biological activities, spanning antibacterial, anti-inflammatory, antitumor, antioxidant, and neuroprotective properties. These revelations have not only broadened the horizons of natural drug discovery but also presented promising candidates or lead compounds for addressing an array of medical conditions. Notably, their antitumor potential has emerged as a focal point of research, showcasing immense promise in the realm of cancer therapy. Furthermore, their antioxidant and neuroprotective capabilities offer innovative strategies for combating neurodegenerative disorders.

Despite the notable progress made in elucidating the distribution, structural features, and biological activities of xanthone dimers, the journey ahead is fraught with both challenges and opportunities. Future research endeavors should prioritize several fronts: firstly, unraveling the intricacies of their biosynthetic pathways and harnessing synthetic biology tools for efficient production; secondly, reinforcing structure–activity relationship studies to discern the molecular underpinnings of their biological effects; thirdly, conducting rigorous pharmacological and toxicological assessments to establish a scientific basis for clinical translation; and lastly, exploring their potential applications beyond medicine, such as in food and cosmetics, to expand their market reach and value.

In essence, xanthone dimers, sourced from plants, fungi, and lichens, represent a class of natural products brimming with research significance. They have enriched our comprehension of chemical diversity in nature and ignited new hopes and challenges in the domains of novel drug development, disease management, and healthcare. With relentless advancements in research technologies and intensified interdisciplinary collaboration, it is anticipated that this field will continue to yield groundbreaking discoveries and transformative achievements in the years to come.

## Figures and Tables

**Table 1:** The occurrence of xanthone dimers in angiosperms, fungi, and lichens.

Division	Class	Family	Genus	Number of Species
Plant				
Angiosperm	Dicotyledoneae	Clusiaceae	*Garcinia*, *Mesua*, *Symphonia*, *Cratoxylum*, *Caraipa*	13
		Hypericaceae	*Hypericum*	4
		Calophyllaceae	*Calophyllum*	1
		Anacardiaceae	*Mangifera*	1
		Gentianaceae	*Swertia*, *Gentianella*	4
Lichen	Basidiolichenes	Ramalinaceae	*Usnea*	2
Fungi				
Ascomycota	Eurotiomycetes	Trichocomaceae	*Aspergillus*, *Talaromyces*	14
	Sordariomycetes	Diaporthaceae	*Diaporthe*, *Aschersonia*	2
		Sordariaceae	*Neurospora*	1
	Leotiomycetes	Dermateaceae	*Cryptosporiopsis*	1
	Ascomycetes	Leptosphaeriaceae	*Subplenodomus*	1
	Dothideomycetes	Muyocopronaceae	*Muyocopron*	1
		Blennoriaceae	*Blennoria.*	1
		Phaeosphaeriaceae	*Setophoma*	1
Ascomycotina	Plectomycetes	Eurotiaceae	*Penicillium*	8
	Pyrenomycetes	Clavicipitaceae	*Claviceps*	1
Basidiomycota	Agaricomycetes	Peniophoraceae	*Peniophora*	1
Deuteromycotina	Coelomycetes	Sphaeropsidales	*Phoma*, *Phomopsis*	9
		Incertae sedis	*Pyrenochaeta*	1
	Dothidea	Dermateaceae	*Alternaria*, *Cladosporium*	2
	Hyphomycetes	Tubeufiaceae	*Monodictys*	1
		Moniliaceae	*Paecilomyces*	2
Other fungi	Ascomycete IBWF11-95A			1
	PM0651480 (isolated as an endophytic fungus from the leaves of *Mimosops elengil*)			1
	Non-sporulating fungus, MF6460 (isolated from leaf litter of *Manilkara bidentata*)			1
	Mangrove endophytic fungus No. ZSU44			1

**Table 2:** Xanthone dimers (the letters a, b, c, d, e, and f represent xanthone and its four subcategories).

Number	Xanthone Type	Monomer Type	Subtype	Compound Name	Molecular Formula	Source (Plant Part)	Ref.
1	Xanthone (a)	a–a	C1–C1’	Hyperidixanthone	C_30_H_22_O_10_	*Hypericum chinense* (entire plant)	[29]
2			C2–C2’	Swertiabixanthone I	C_32_H_23_O_17_	*Swertia franchetiana* (entire plant)	[30]
3				Swertiabixanthone II	C_27_H_16_O_12_	*Swertia pseudochinensis* (entire plant)	[31]
4				Swertiabisxanthone I 8′-*O*-D-glucopyranoside	C_32_H_24_O_17_	*Swertia punicea*, *Gentianella amarella* ssp. acuta (entire plant)	[23,32]
5			C2–C4’	Swertipunicoside	C_33_H_26_O_17_	*S. franchetiana* (entire plant)	[33]
6				3-*O*-demethylswertipunicoside	C_32_H_24_O_17_	*S. franchetiana* (entire plant)	[23]
7			C4–C2’	Puniceaside A	C_32_H_24_O_17_	*S. punicea* (entire plant)	[23]
8			C4–C4’	Mangiferoxanthone A	C_38_H_34_O_22_	*Mangifera indica* (stem bark)	[34]
9			C8–C8’	Ploiarixanthone	C_26_H_14_O_9_	*Ploiartium alternifolium* (branches)	[35]
10			Complex	Bigarciculenxanthone A	C_46_H_46_O_13_	*Garcinia esculenta* (twigs and leaves)	[36]
11				Bigarciculenxanthone B	C_46_H_46_O_13_	*G. esculenta* (twigs and leaves)	[36]
12				Bigarciculenxanthone C	C_46_H_44_O_12_	*G. esculenta* (twigs and leaves)	[36]
13				Bigarcinenone B	C_36_H_28_O_11_	*Garcinia xanthochymus* (bark)	[37]
14				Biscaloxanthone	C_47_H_48_O_10_	*Calophyllum canum* (stem bark)	[38]
15				Globulixanthone E	C_37_H_30_O_9_	*Symphonia globulifera* (root bark)	[39]
16				Garcinoxanthone A	C_48_H_50_O_13_	*Garcinia mangostana* (pericarp)	[40]
17				Garcinoxanthone B	C_48_H_50_O_13_	*G. mangostana* (pericarp)	[40]
18				Garcinoxanthone C	C_48_H_47_O_13_	*G. mangostana* (pericarp)	[40]
19				Cratoxyxanthone	C_48_H_50_O_13_	*G. mangostana* (bark)	[41]
20				Bigarcinenone A	C_56_H_62_O_13_	*G. xanthochymus* (bark)	[22,42]
21				Bixanthone C	C_31_H_21_O_12_	*Hypericum japonicum* (entire plant)	[27]
22				Bixanthone D	C_36_H_29_O_12_	*H. japonicum* (entire plant)	[27]
23				Bijaponicaxanthone	C_36_H_28_O_13_	*H. japonicum*, *Hypericum henryi* (aerial parts)	[43]
24				Jacarelhyperol A	C_36_H_28_O_13_	*H. japonicum* (aerial parts)	[44]
25				Jacarelhyperol B	C_36_H_28_O_12_	*H. japonicum* (aerial parts)	[44]
26				Jacarelhyperol D	C_31_H_21_O_13_	*H. japonicum* (aerial parts)	[45]
27				Bijaponicaxanthone C	C_36_H_30_O_13_	*H. japonicum*, *Hypericum riparium* (entire plant)	[45]
28				Griffipavixanthone	C_36_H_28_O_12_	*Garcinia griffithii*, *Garcinia pavifolia* (bark), *G. esculenta* (twigs and leaves), *Garcinia oblongifolia* (bark)	[14,15,25,46]
29				Garmoxanthone	C_36_H_28_O_12_	*G. mangostana* (pericarp)	[20]
30				Garcilivin A	C_36_H_28_O_10_	*Garcinia livingstonei* (bark)	[47,48]
31				Garcilivin C	C_36_H_28_O_10_	*G. livingstonei* (bark)	[47,48]
32				Garciobioxanthone	C_36_H_32_O_13_	*G. oblongifolia* (bark)	[49]
33				Mesuabixanthone A	C_33_H_24_O_12_	*Mesua ferrea* (stem bark)	[50]
34				Mesuabixanthone B	C_34_H_26_O_12_	*M. ferrea* (stem bark)	[50]
35				Mesuferrol A	C_32_H_22_O_12_	*M. ferrea* (bark)	[51]
36				Mesuferrol B	C_33_H_24_O_12_	*M. ferrea* (bark)	[51]
37				Schomburgkixanthone	C_36_H_34_O_14_	*Garcinia schomburgkiana* (twigs)	[52]
38				Garcilivin B	C_36_H_28_O_10_	*G. livingstonei* (bark)	[48]
39		a–a	C–O–C	Austradixanthone	C_30_H_22_O_13_	*Aspergillus austroafricanus*	[53]
40		a–a	C–S–C	Castochrin	C_32_H_21_O_12_	*Alternaria* sp.	[54]
41	Xanthone (a)-tetrahydroxanthone (e)	a–e	C2–C2’	Puniceaside B	C_32_H_28_O_17_	*S. punicea* (entire plant)	[23]
42		a–e	C–NH–C	Incarxanthone F	C_30_H_23_NO_12_	*Peniophora incarnata* Z4	[55]
43	Dihydroxanthones (b/c)	b–b	C2–C2’	Subplenone C	C_30_H_26_O_10_	*Subplenodomus* sp. CPCC 401465	[56]
44			C2–C2’	Phomalevone A	C_30_H_26_O_10_	*Phoma* sp.	[57]
45			C2–C2’	Subplenone D	C_30_H_24_O_10_	*Subplenodomus* sp. CPCC 401465	[56]
46			C2–C2’	Subplenone E	C_30_H_24_O_10_	*Subplenodomus* sp. CPCC 401465	[56]
47			C2–C2’	Subplenone F	C_30_H_22_O_10_	*Subplenodomus* sp. CPCC 401465	[56]
48			C2–C2’	Phomalevone C	C_30_H_24_O_10_	*Phoma* sp.	[57]
49			C2–C2’	Subplenone I	C_30_H_26_O_11_	*Subplenodomus* sp. CPCC 401465	[56]
50		b–d	C2–C2’	Subplenone G	C_30_H_28_O_10_	*Subplenodomus* sp. CPCC 401465	[56]
51			C2–C4’	Subplenone A	C_30_H_24_O_10_	*Subplenodomus* sp. CPCC 401465	[56]
52			C2–C4’	Subplenone B	C_30_H_24_O_10_	*Subplenodomus* sp. CPCC 401465	[56]
53		c–e	3,4-epoxy	Terricoxanthone A	C_32_H_26_O_15_	*Neurospora terricola* HDF-Br-2	[58]
54			3,4-epoxy	Terricoxanthone B	C_32_H_26_O_15_	*N. terricola* HDF-Br-2	[58]
55			3,4-epoxy	Terricoxanthone C	C_32_H_26_O_15_	*N. terricola* HDF-Br-2	[58]
56			3,4-epoxy	Terricoxanthone D	C_32_H_26_O_14_	*N. terricola* HDF-Br-2	[58]
57			3,4-epoxy	Terricoxanthone E	C_32_H_26_O_14_	*N. terricola* HDF-Br-2	[58]
58	Tetrahydroxanthones (d/e)	e–e	Complex	Terricoxanthone F	C_32_H_28_O_15_	*N. terricola* HDF-Br-2	[58]
59		d–d	C2–C2’	Secaionic acid A (Ergochrome AA)	C_32_H_30_O_14_	*Pyrenochaeta terrestris*, *Penicillium chrysogenum* C-7-2-1	[59,60,61]
60			C2–C2’	Secaionic acid B (Ergochrome BB)	C_32_H_30_O_14_	*Claviceps purpurea*	[12,62,63]
61			C2–C2’	Secaionic acid C (Ergochrome AB)	C_32_H_30_O_14_	*C. purpurea*	[64]
62			C2–C2’	Secalonic acid D (Ergochrome EE)	C_32_H_30_O_14_	*Penicillium* sp. F11, *Penicillium oxalicum*, *Aspergillus aculeatus*, *Aspergillus aculeatinus* WHUF0198, *Aspergillus* sp. TPU1343, *C. purpurea* the mangrove endophytic fungus No. ZSU44	[12,16,17,19,62]
63			C2–C2’	Secalonic acid E (Ergochrome FF)	C_32_H_30_O_14_	*C. purpurea*, *P. terrestris*	[60,61,63,65]
64			C2–C2’	Secalonic acid F (Ergochrome BE)	C_32_H_30_O_14_	*Aspergillus* sp. TPU1343, *P. chrysogenum* C-7-2-1, *A. aculeatus*	[17,59,60,62,66,67]
65			C2–C2’	Secalonic acid G (Ergochrome AG)	C_32_H_30_O_14_	*P. terrestris*	[61]
66			C2–C2’	Diaporxanthone F	C_32_H_32_O_13_	*Diaporthe goulteri* L17	[68]
67			C2–C2’	Phomoxanthone E	C_34_H_34_O_14_	*Phomopsis* sp. xy21; *D. goulteri* L17	[68,69]
68			C2–C2’	Dicerandrol A	C_34_H_34_O_14_	*Phomopsis longicolla*, *Phomopsis* sp. xy21, *Phomopsis* sp. PSU-D15; *Phomopsis asparagi* DHS-48 and *Phomopsis* sp. DHS-11	[70,71,72,73]
69			C2–C2’	Dicerandrol B	C_36_H_36_O_15_	*P. longicolla*, *Phomopsis* sp. xy21; *Paecilomyces* sp, *Paecilomyces* sp. EJC01.1	[13,68,71,72,74]
70			C2–C2’	Dicerandrol C	C_38_H_38_O_16_	*Phomopsis* sp. xy21; *P. asparagi* DHS-48 and *Phomopsis* sp. DHS-11	[71,72]
71			C2–C2’	Hirtusneanoside	C_40_H_46_O_17_	*Usnea hirta* (whole thallus)	[75]
72			C2–C2’	Hirtusneanine	C_34_H_36_O_13_	*U. hirta* (whole thallus)	[75]
73			C2–C2’	Chrysoxanthone H	C_32_H_30_O_14_	*P. chrysogenum* C-7-2-1	[59]
74			C2–C2’	Versixanthone G	C_32_H_31_O_14_	*Aspergillus versicolor* HDN1009	[76]
75			C2–C2’	Versixanthone H	C_32_H_31_O_14_	*A. versicolor* HDN1009	[76]
76			C2–C2’	Ascherxanthone A	C_32_H_34_O_10_	*Aschersonia* sp. BCC 8401	[77]
77			C2–C2’	Ascherxanthone B	C_32_H_34_O_12_	*Aschersonia luteola* BCC 8774	[78]
78			C2–C2’	Muyocoxanthone E	C_32_H_34_O_12_	*Muyocopron laterale*	[79]
79			C2–C2’	Muyocoxanthone F	C_32_H_34_O_12_	*M. laterale*	[79]
80			C2–C2’	Rugulotrosin A	C_32_H_30_O_14_	*Penicillium* sp.	[12,80]
81			C2–C2’	Xanthonol	C_38_H_34_O_14_	Non-sporulating fungus, MF6460 (isolated from leaf litter of *Manilkara bidentata*)	[81]
82			C2–C2’	Cryptosporioptide A	C_38_H_30_O_18_	*Cryptosporiopsis* sp. 8999	[82,83]
83			C2–C2’	Cryptosporioptide B	C_38_H_34_O_18_	*Cryptosporiopsis* sp. 8999	[82]
84			C2–C2’	Cryptosporioptide C	C_40_H_38_O_18_	*Cryptosporiopsis* sp. 8999	[82]
85			C2–C2’	Penicixanthone E	C_38_H_34_O_18_	*Penicillium purpurogenum* SC0070	[84]
86			C2–C2’	Penicixanthone F	C_40_H_38_O_18_	*P. purpurogenum* SC0070	[84]
87			C2–C2’	Penicixanthone G	C_39_H_36_O_18_	*P. purpurogenum* SC0070	[84]
88			C2–C2’	Penicixanthone H	C_35_H_32_O_15_	*P. purpurogenum* SC0070	[84]
89			C2–C2’	Aflaxanthone A	C_30_H_30_O_11_	*Aspergillus flavus* QQYZ	[18]
90			C2–C2’	Aflaxanthone B	C_30_H_30_O_11_	*A. flavus* QQYZ	[18]
91		d–d	C4–C4’	Talaroxanthone (4-4’-secalonic acid E)	C_32_H_30_O_14_	*Talaromyces* sp.	[85,86]
92			C4–C4’	Versixanthone M	C_32_H_30_O_14_	*A. versicolor* HDN1009	[76]
93			C4–C4’	Chrysoxanthone K	C_32_H_30_O_14_	*P. chrysogenum* C-7-2-1	[59]
94			C4–C4’	Phomoxanthone A	C_38_H_38_O_16_	*Phomopsis* sp. BCC 1323, y254 and IM 41-1; *Paecilomyces* sp. EJC01.1, *D. goulteri* L17	[68,74,87,88]
95			C4–C4’	Deacetylphomoxanthone A	C_30_H_30_O_12_	*Phomopsis* sp. BCC 1323	[87]
96			C4–C4’	12-*O*-deacetylphomoxanthone A	C_36_H_36_O_15_	*P. asparagi* DHS-48 and *Phomopsis* sp. DHS-11; *Phomopsis* sp. IM 41-1	[68,88,89]
97			C4–C4’	Deacetylphomoxanthone C	C_34_H_33_O_14_	*D. goulteri* L17, HNY29-2B	[68,71]
98			C4–C4’	4-4′-secalonic acid A	C_32_H_30_O_14_	*P. chrysogenum* C-7-2-1	[59,85]
99			C4–C4’	4-4’-secalonic acid D	C_32_H_30_O_14_	*A. aculeatinus* WHUF0198	[19,90]
100		d–d	C2–C4’	Chrysoxanthone J	C_32_H_30_O_14_	*P. chrysogenum* C-7-2-1	[59]
101			C2–C4’	Penicillixanthone A (2-4′-linked SAA)	C_32_H_31_O_14_	*Aspergillus niger*, *Aspergillus fumigates*; *P. chrysogenum* C-7-2-1	[17,59,60,91]
102			C2–C4’	Penicillixanthone B	C_32_H_31_O_14_	*Setophoma terrestris* MSX45109	[60,61]
103			C2–C4’	Secalonic acid F1	C_32_H_30_O_14_	*Aspergillus* sp. TPU1343, *P. chrysogenum* C-7-2-1	[17,59,60]
104			C2–C4’	Deacetylphomoxanthone B	C_34_H_34_O_14_	*Phomopsis* sp. BCC 1323, *Phomopsis* sp. PSU-D15	[70,71]
105			C2–C4’	Phomoxanthone B	C_38_H_38_O_16_	*Phomopsis* sp. BCC 1323 and xy21	[70,74,87,92]
106			C2–C4’	Penexanthone A	C_36_H_36_O_15_	*Penicillium* sp. CR1642D, *D. goulteri* L17	[68,71,93]
107			C2–C4’	Versixanthone J	C_32_H_31_O_15_	*A. versicolor* HDN1009	[76]
108			C2–C4’	Versixanthone K	C_32_H_31_O_15_	*A. versicolor* HDN1009	[76]
109			C2–C4’	Versixanthone L	C_32_H_30_O_14_	*A. versicolor* HDN1009	[76]
110			C2–C4’	Neosartorin	C_34_H_32_O_15_	*Aspergillus novofumigatus*	[80,94]
111			C2–C4’	Deacetylneosartorin	C_32_H_28_O_14_	*A. novofumigatus*	[94]
112			C2–C4’	Novofumigatin A	C_30_H_26_O_14_	*A. novofumigatus*	[94]
113			C2–C4’	Rugulotrosin B	C_32_H_30_O_14_	*Penicillium* sp.	[80]
114			C2–C4’	Chrysoxanthone I	C_32_H_30_O_14_	*P. chrysogenum* C-7-2-1	[59]
115			C2–C4’	Eumitrin H	C_34_H_34_O_14_	*Usnea baileyi* (whole thallus)	[95]
116		d–d	C–O–C	Asperdichrome	C_32_H_34_O_14_	*Aspergillus* sp. TPU1343	[62]
117	Tetrahydroxanthone (d) and hexahydroxanthone (f)	d–f	C2–C2’	Ergochrome AD	C_32_H_32_O_15_	*C. purpurea*	[64]
118			C2–C2’	Ergochrome BD	C_32_H_32_O_15_	*C. purpurea*	[64]
119			C2–C2’	Ergochrome DD	C_31_H_28_O_14_	*C. purpurea*	[64]
120			C2–C2’	Eumitrin C	C_32_H_32_O_13_	*U. baileyi* (whole thallus)	[96]
121			C2–C2’	Ergochrysin A (Ergochrome AC)	C_31_H_28_O_14_	*C. purpurea*	[64]
122			C2–C2’	Ergochrysin B (Ergochrome BC)	C_31_H_28_O_14_	*C. purpurea*	[64]
123			C2–C2’	Subplenone H	C_30_H_28_O_11_	*Subplenodomus* sp. CPCC 401465	[56]
124			C4–C2’	Aculeaxanthone E	C_32_H_30_O_15_	*A. aculeatinus* WHUF0198	[18]
125			C4–C2’	Eumitrin A_1_	C_34_H_32_O_15_	*U. baileyi* (whole thallus)	[97]
126			C4–C2’	Eumitrin A_2_	C_34_H_34_O_14_	*U. baileyi* (whole thallus)	[97]
127			C4–C2’	Eumitrin B	C_34_H_34_O_14_	*U. baileyi* (whole thallus)	[97]
128			C4–C2’	Eumitrin T	C_34_H_34_O_14_	*U. baileyi* (whole thallus)	[27]
129		d–f	2,3-ring	Nidulaxanthone A	C_32_H_28_O_12_	*Aspergillus* sp. F029	[98]
130	Hexahydroxanthones (f)	f–f	C2–C2’	Eumitrin F	C_32_H_34_O_12_	*U. baileyi* (whole thallus)	[95]
131			C2–C2’	Eumitrin G	C_32_H_34_O_12_	*U. baileyi* (whole thallus)	[95]
132			C2–C2’	Phomoxanthone C	C_30_H_34_O_14_	*Phomopsis* sp.	[69]
133			C2–C2’	Phomoxanthone D	C_30_H_32_O_14_	*Phomopsis* sp. xy21, *P. asparagi* DHS-48 and *Phomopsis* sp. DHS-11	[69,89]
134			C2–C2’	Cladoxanthone B	C_37_H_34_O_12_	*Cladosporium* sp.	[99]
135			C2–C2’	Ergochrome CD	C_31_H_30_O_15_	*C. purpurea*	[64]
136			C2–C2’	Ergoflavin (Ergochrome CC)	C_32_H_34_O_16_	*C. purpurea*, PM0651480 (isolated as an endophytic fungus from the leaves of *Mimosops elengi*)	[64,100]
137		f–f	C2–C4’	Eumitrin E	C_33_H_31_O_15_	*U. baileyi* (whole thallus)	[96]
138	Heterodimers	Xanthone–flavone		Swertifrancheside	C_35_H_29_O_17_	*S. franchetiana* (entire plant)	[33]
139		Xanthonelignans		Cadensin A	C_24_H_20_O_9_	*Caraipa densiflora*	[101]
140				Cadensin B	C_25_H_22_O_10_	*C. densiflora*	[101]
141				Kielcorin	C_24_H_20_O_8_	*Psorospermum febrifugum* (root)	[102]
142				Cadensin D	C_25_H_22_O_9_	*P. febrifugum* (root)	[102]
143				Isocadensin D	C_25_H_22_O_9_	*P. febrifugum* (root)	[102]
144				Isodensin D monoacetate	C_27_H_24_O_10_	*P. febrifugum* (root)	[102]
145				Cadensin F	C_26_H_24_O_10_	*P. febrifugum* (root)	[102]
146				6-Hydroxyisocadensin F	C_26_H_24_O_11_	*P. febrifugum* (root)	[102]
147				Cadensin G	C_24_H_20_O_10_	*P. febrifugum* (root)	[102]
148				5’-Demethoxycadensin G	C_23_H_18_O_9_	*Cratoxylum cochinchinense* (bark)	[41]
149				(±) Esculentin A	C_24_H_20_O_10_	*G. esculenta* (branches)	[42]
150	Heterodimers	Xanthone– benzophenones		Garciduol A	C_27_H_18_O_9_	*Garcinia duicis* (root)	[103]
151				Garciduol B	C_27_H_18_O_10_	*G. duicis* (root)	[103]
152				Garciduol C	C_27_H_18_O_9_	*G. duicis* (root)	[103]
153			C–S–C	Dioschrin	C_33_H_26_O_13_S	*Alternaria* sp.	[54]
154				Subplenone J	C_30_H_26_O_10_	*Subplenodomus* sp. CPCC 401465	[56]
155				Secalonic acid H	C_32_H_28_O_14_	*Aspergillus brunneoviolaceus* MF180246, *P. oxalicurn*	[17,104]
156				Versixanthone I	C_32_H_28_O_14_	*A. versicolor* HDN1009	[76]
157				Secalonic acid I	C_32_H_28_O_14_	*P. oxalicurn*	[104]
158			C–O–C	Chrysoxanthone	C_32_H_30_O_14_	The ascomycete IBWF11-95A; *Aspergillus* sp. TPU1343	[62,105]
159		d–chromanone	C2–C2’	Chrysoxanthone B	C_32_H_30_O_14_	*A. aculeatinus* WHUF0198	[19]
160			C2–C2’	Chrysoxanthone C	C_32_H_30_O_14_	*A. aculeatinus* WHUF0198, *A. brunneoviolaceus* MF180246	[17,19]
161			C2–C2’	(-)-Blennolide G	C_32_H_30_O_10_	*Blennoria* sp.	[54]
162			C2–C2’	Blennolide G	C_32_H_30_O_10_	*Blennoria* sp.	[106]
163			C2–C2’	Muyocoxanthone G	C_32_H_34_O_11_	*M. laterale*	[79]
164			C2–C2’	Muyocoxanthone H	C_32_H_34_O_12_	*M. laterale*	[79]
165			C2–C2’	Muyocoxanthone J	C_32_H_34_O_11_	*M. laterale*	[79]
166			C2–C2’	Muyocoxanthone K	C_32_H_34_O_11_	*M. laterale*	[79]
167			C2–C2’	Versixanthone D	C_32_H_30_O_14_	*A. versicolor* HDN1009	[16]
168			C2–C2’	Versixanthone F	C_33_H_34_O_15_	*A. versicolor* HDN1009	[16]
169			C2–C2’	Chrysoxanthone E	C_32_H_30_O_14_	*P. chrysogenum* C-7-2-1	[59]
170			C2–C2’	Diaporxanthone A	C_32_H_32_O_13_	*D. goulteri* L17	[68]
171			C2–C2’	Diaporxanthone B	C_32_H_32_O_13_	*D. goulteri* L17	[68]
172			C2–C2’	Phomolactonexanthone B	C_34_H_34_O_14_	*D. goulteri* L17, *Phomopsis* sp. HNY29-2B	[71]
173			C2–C2’	Diaporxanthone C	C_36_H_38_O_16_	*D. goulteri* L17	[68]
174			C2–C2’	Diaporxanthone D	C_36_H_38_O_16_	*D. goulteri* L17	[68]
175			C4–C4’	Versixanthone B	C_32_H_30_O_14_	*A. versicolor* HDN1009	[16]
176			C4–C4’	Diaporxanthone E	C_32_H_32_O_13_	*D. goulteri* L17	[68]
177			C2–C4’	Chrysoxanthone D	C_32_H_30_O_14_	*P. chrysogenum* C-7-2-1	[59]
178			C2–C4’	Versixanthone C	C_32_H_30_O_14_	*A. versicolor* HDN1009	[16]
179			C2–C4’	Phomolactonexanthone A	C_34_H_33_O_14_	*D. goulteri* L17, *Phomopsis* sp. HNY29-2B	[68,71]
180			C2–C4’	Muyocoxanthone I	C_32_H_34_O_12_	*M. laterale*	[79]
181			C2–C4’	Muyocoxanthone L	C_32_H_34_O_11_	*M. laterale*	[79]
182			C4–C2’	Aculeaxanthone A	C_32_H_30_O_14_	*A. aculeatinus* WHUF0198	[19]
183			C4–C2’	Chrysoxanthone F	C_32_H_30_O_14_	*P. chrysogenum* C-7-2-1	[59]
184			C4–C2’	Chrysoxanthone G	C_32_H_30_O_14_	*P. chrysogenum* C-7-2-1	[59]
185			C4–C2’	Versixanthone A	C_32_H_30_O_14_	*A. versicolor* HDN1009	[16]
186			C4–C2’	Versixanthone E	C_33_H_34_O_15_	*A. versicolor* HDN1009	[16]
187		f–chromanone	C2–C2’	Eumitrin I	C_32_H_32_O_13_	*U. baileyi* (whole thallus)	[107]
188			C2–C2’	Eumitrin J	C_32_H_32_O_13_	*U. baileyi* (whole thallus)	[107]
189			C2–C2’	Phomoxanthone N	C_30_H_30_O_13_	*P. asparagi* DHS-48 and *Phomopsis* sp. DHS-11	[89]
190			C2–C2’	Ergoxantin	C_31_H_28_O_14_	Portuguese ergot drug	[108]
191			C2–C2’	Phomoxanthone L	C_30_H_30_O_13_	*P. asparagi* DHS-48 and *Phomopsis* sp. DHS-11	[89]
192			C2–C2’	Phomoxanthone M	C_30_H_30_O_13_	*P. asparagi* DHS-48 and *Phomopsis* sp. DHS-11	[89]
193			C2–C4’	Eumitrin K	C_32_H_32_O_13_	*U. baileyi* (whole thallus)	[107]
194			C4–C2’	Eumitrin D	C_34_H_34_O_14_	*U. baileyi* (whole thallus)	[96]
195		chromanones	C2–C2’	Aculeaxanthone B	C_32_H_30_O_14_	*A. aculeatinus* WHUF0198	[19]
196			C2–C2’	Aculeaxanthone C	C_32_H_30_O_14_	*A. aculeatinus* WHUF0198	[19]
197			C2–C2’	Phomopsis-H76 A/Diaporthochromone B	C_30_H_30_O_12_	*P. asparagi* DHS-48 and *Phomopsis* sp. DHS-11, *Phomopsis* sp. (#zsu-H76)	[65,89,109]
198			C2–C2’	Blennolide H	C_32_H_30_O_14_	*Alternaria* sp.	[54]
199			C2–C2’	Blennolide I	C_32_H_30_O_14_	*Blennoria* sp.	[54]
200			C2–C2’	Muyocoxanthone A	C_32_H_34_O_12_	*M. laterale*	[79]
201			C2–C2’	Muyocoxanthone B	C_32_H_34_O_12_	*M. laterale*	[79]
202			C2–C2’	Muyocoxanthone C	C_32_H_34_O_12_	*M. laterale*	[79]
203			C2–C2’	Monodictyochrome B	C_30_H_30_O_11_	*Monodictys putredinis*	[110]
204			C2–C2’	Aculeaxanthone D	C_33_H_34_O_15_	*A. aculeatinus* WHUF0198	[18]
205			C2–C2’	Chrysoxanthone O	C_32_H_32_O_15_	*P. chrysogenum* C-7-2-1	[59]
206			C2–C2’	Chrysoxanthone P	C_32_H_34_O_15_	*P. chrysogenum* C-7-2-1	[59]
207			C2–C2’	Paecilins M	C_32_H_34_O_15_	*P. chrysogenum* C-7-2-1	[59]
208			C2–C2’	Chrysoxanthone L	C_32_H_34_O_16_	*P. chrysogenum* C-7-2-1	[59]
209			C2–C4’	Muyocoxanthone D	C_32_H_34_O_12_	*M. laterale*	[79]
210			C2–C4’	Chrysoxanthone N	C_32_H_32_O_15_	*P. chrysogenum* C-7-2-1	[59]
211			C4–C2’	Blennolide J	C_32_H_30_O_14_	*Blennoria* sp.	[54]
212			C4–C2’	Monodictyochrome A	C_30_H_30_O_11_	*M. putredinis*	[110]
213			C4–C2’	Chrysoxanthone M	C_32_H_34_O_16_	*P. chrysogenum* C-7-2-1	[59]
214			C4–C2’	Chrysoxanthone Q	C_32_H_34_O_15_	*P. chrysogenum* C-7-2-1	[59]

**Table 3:** Bioactivity studies of xanthone dimers.

Compounds	Biological Activities	Testing Subjects	Outcome	Effects/Mechanisms	Ref.
Swertiabisxanthone-I 8′-*O*-D-glucopyranoside (**4**)	Neuroprotective	H_2_O_2_-induced PC12 cell	Cell viability up to 157.8% ± 6.0 (at a concentration of 25 μg/mL)	—	[23]
Swertipunicoside (**5**)	Antiviral	Inhibitor of HIV reverse transcriptase	ED_50_ = 3.0 μg/mL	—	[33]
3-*O*-Demethylswertipunicoside (**6**)	Neuroprotective	H_2_O_2_-induced PC12 cell	Cell viability up to 123.0% ± 5.6 (at a concentration of 25 μg/mL)	Increase the protein expression of both tyrosine hydroxylase (TH) and DJ-1	[23,24]
Mangiferoxanthone A (**8**)	Anti-influenza	Influenza neuraminidase (NA)	55.8% inhibition	—	[34]
	Antiviral	Coxsackie virus B3 3C protease	46.1% inhibition at 100 μM		[34]
Bigarciculenxanthone A (**10**)	Cytotoxicity	Myeloid leukemia HL-60, lung cancer A-549, hepatocellular carcinoma SMMC-7721, breast cancer MDA-MB-231, colon cancer SW480 cells	IC_50_ = 15.2–22.9 μM	—	[36]
Bigarciculenxanthone B (**11**)	Cytotoxicity	HL-60, A-549, SMMC-7721, MDA-MB-231, SW480 cells	IC_50_ = 17.8–29.9 μM	—	[36]
Bigarcinenone B (**13**)	Antioxidative	1,1-diphenyl-2-picrylhydrazyl (DPPH) free-radical-scavenging method	IC_50_ = 20.14 μM	—	[37]
		Luminol-H_2_O_2_-Co^II^-EDTA luminescence system	IC_50_ = 2.85 μM	—	[37]
Biscaloxanthone (**14**)	Cytotoxicity	Human lung carcinoma (A549), breast cancer (MCF-7), cervical cancer (C33A), and normal rat fibroblast (3T3L1)	IC_50_ > 50 μM	—	[38]
Globulixanthone E (**15**)	Antimicrobial	*Staphylococcus aureus* (ATCC 6538) *Bacillus subtilis* (ATCC 6633) *Vibrio anguillarium* (ATCC 19264)	MIC = 3.12–5.56 μg/mL	—	[39]
Garcinoxanthone B (**17**)	Anti-inflammation	Lipopolysaccharide (LPS)-stimulated RAW264.7 cells	IC_50_ = 11.3 ± 1.7 μM	Inhibit production of NO	[40]
Garcinoxanthone C (**18**)	Anti-inflammation	As above	IC_50_ = 18.0 ± 1.8 μM	Inhibit production of NO	[40]
Cratoxyxanthone (**19**)	Cytotoxicity	K562, HeLa cells	IC_50_ = 17.1–39.8 μg/mL	—	[112]
Bigarcinenone A (**20**)	Antioxidative	DPPH radical-scavenging test	IC_50_ = 9.2 μM	—	[22]
Bixanthone C (**21**)	Treat hepatic brosis	—	—	—	[27]
Bixanthone D (**22**)	Treat hepatic brosis	—	—	—	[27]
Jacarelhyperol A (**24**)	Anti-PAF	Male ddY mice (SPF grade), 6 weeks old	—	Inhibit PAF-induced hypotension in vivo	[44]
Jacarelhyperol B (**25**)	Anti-PAF	As above	—	As above	[44]
Griffipavixanthone (**28**)	Anticancer	Human non-small-cell lung cancer H520 cell	IC_50_ = 3.03 ± 0.21 μM	Induce cell apoptosis through mitochondrial apoptotic pathway accompanying with ROS production	[25]
		Human esophageal cancer cell line TE1 and KYSE150 cells	—	Inhibit cell migration and invasion; render cell proliferation and induce G2/M cell cycle arrest; inhibit tumor metastasis and proliferation via downregulating RAF–MEK–ERK pathway	[15]
		Human breast cancer cells MCF-7 and T-47D	IC_50, 48h_ = 9.64 ± 0.12 μM IC_50, 48h_ = 10.21 ± 0.38 μM	Increase the mRNA and protein expression level of p53 and its target genes, upregulate p53 and Bax expression, suppress Bcl-2 expression, induce MCF-7 cell apoptosis	[14]
	Hypoglycemic	Sucrase	IC_50_ = 4.58 μM	Inhibit sucrase	[52]
	XO inhibitors	Xanthine oxidase	IC_50_ = 6.3 μM	Inhibit XO	[42]
Garmoxanthone (**29**)	Antibacterial	MRSA (ATCC 43300) MRSA (CGMCC1.12409)	MIC = 3.9 μg/mL	—	[20]
		*Vibrio rotiferianus* (MCCC E385), *V. vulnificus* (MCCC E1758), *V. campbellii* (MCCC E333)	MIC = 15.6–31.2 μg/mL	—	[20]
Garcilivin A (**30**)	Cytotoxicity	MRC-5 cells	IC_50_ = 2.0 μM	—	[47]
	Antiparasitic	*Trypanosoma brucei brucei Trypanosoma cruzi* *Plasmodium falciparum*	IC_50_ = 0.4 μM IC_50_ = 4.0 μM IC_50_ = 6.7 μM	—	[47]
Garcilivin C (**31**)	Cytotoxicity	MRC-5 cells	IC_50_ = 52.3 μM	—	[47]
	Antiparasitic	*T. cruzi*	IC_50_ = 7.7 μM	—	[47]
Schomburgkixanthone (**37**)	Hypoglycemic	In vitro inhibition rat intestinal a-glucosidase (maltase, sucrase)	IC_50_ = 0.79 μΜ IC_50_ = 0.81 μΜ	—	[52]
Puniceaside B (**41**)	Neuroprotective	H_2_O_2_-induced PC12 cell	Cell viability 98.1% ± 6.8 at a concentration of 25 μg/mL	—	[23]
Subplenone C (**43**)	Antibacterial	Methicillin-resistant *S. aureus* ATCC 43300 and 700698, *S. aureus* ATCC 29213 MSSA, vancomycin-resistant *Enterococcus faecium* ATCC 700221, and *Staphylococcus epidermidis* ATCC 12228 MSSE	MIC = 1.0–4.0 μg/mL	—	[56]
Phomalevone A (**44**)	Antibacterial	*B. subtilis* (ATCC 6051) and *S. aureus* (ATCC 29213)	Inhibitory zones of 36 and 23 mm	—	[57]
Subplenone D (**45**)	Antibacterial	Methicillin-resistant *S. aureus* ATCC 43300 and 700698, *S. aureus* ATCC 29213 MSSA, vancomycin-resistant *E. faecalis* ATCC 51299, vancomycin-resistant *Enterococcus faecium* ATCC 700221, *E. faecalis* ATCC 29212 VSE, and *S. epidermidis* ATCC 12228 MSSE	MIC = 0.125–4.0 μg/mL	—	[56]
Subplenone E (**46**)	Antibacterial	As above	MIC = 0.125–2.0 μg/mL	—	[56]
Subplenone F (**47**)	Antibacterial	As above	MIC = 0.25–4.0 μg/mL	—	[56]
Phomalevone C (**48**)	Antibacterial	*B. subtilis* (ATCC 6051) and *S. aureus* (ATCC 29213)	Inhibitory zones of 34 and 22 mm	—	[57]
	Antifungal	*Aspergillus flavus* (NRRL 6541) and *Fusarium verticillioides* (NRRL 25457)	Inhibition zones of 10 mm IC_50_ = 4 μg/mL against *A. flavus*, MIC = 10 μg/mL against *F. verticillioides*	—	[57]
Subplenone I (**49**)	Antibacterial	Methicillin-resistant *S. aureus* ATCC 43300 and 700698, *S. aureus* ATCC 29213 MSSA, vancomycin-resistant *E. faecalis* ATCC 51299, vancomycin-resistant *E. faecium* ATCC 700221, *E. faecalis* ATCC 29212 VSE, and *S. epidermidis* ATCC 12228 MSSE	MIC = 0.5–16 μg/mL	—	[56]
Subplenone G (**50**)	Antibacterial	As above	MIC = 0.125–4.0 μg/mL	—	[56]
Subplenone A (**51**)	Antibacterial	As above	MIC = 0.125–2.0 μg/mL	—	[56]
Subplenone B (**52**)	Antibacterial	As above	MIC = 0.25–4.0 μg/mL	—	[56]
Terricoxanthone F (**58**)	Antibacterial	*Candida Albicans*	MIC = 16 μg/mL	—	[58]
Secaionic acid A (**59**)	Cytotoxicity	H23 human non-small-cell lung carcinoma cells	IC_50_ = 2.6 μM	—	[59]
		MDA-MB-435 (melanoma) and SW-620 (colon) cancer cell lines	IC_50_ = 0.16 μM IC_50_ = 0.41 μM	—	[61]
	Antibacterial	Gram-positive bacterium *Micrococcus luteus* and *S. aureus*	MIC = 38 μg/mL MIC = 75 μg/mL	—	[61]
	Neuroprotective (reduced colchicine cytotoxicity)	Newborn Sprague–Dawley rats	SAA at doses of 3 and 10 μM	Inhibit phosphorylation of JNK and p38 MAPKs, calcium influx, and the activation of caspase-3	[113]
	Neuroprotective	Pregnant Sprague–Dawley rats (14–16 days) and male C57BL/6J mice (8–10 weeks old, 20–22 g)	SAA at doses of 0.15 μg/kg and 0.75 μg/kg	Inhibit the phosphorylation of JNK and p38 MAPK, downregulate Bax expression, and suppress caspase-3 activation	[114]
	Antibacterial	All multidrug-resistant bacterial strains *Escherichia coli* 942, *E. coli* 4814, *S. aureus* 931, *S. aureus* 934, *S. aureus* MRSA 1872, and *K. pneumonia* 815	MIC = 4.7–37.5 μg/mL	—	[115]
Secaionic acid B (**60**)	Antifungal	*Microbotryum violaceum*	13 mm (Agar diffusion assays)	—	[106]
	Antialgal	*Chlorella fusca*	5 mm (Agar diffusion assays)	—	[106]
	Antimicrobial	Gram-positive (*Bacillus megaterium*) and Gram-negative (*E. coli*) bacteria	0 mm and 15 mm (Agar diffusion assays)	—	[106]
Secalonic acid D (**62**)	Cytotoxicity	HL60, K562 cells	IC_50_ = 0.38 μmol/L IC_50_ = 0.43 μmol/L	Induce leukemia cell apoptosis and cell cycle arrest of G1 with involvement of GSK-3β/β-catenin/c-Myc pathway	[116]
		BGC-823, SKHEP, SGC-7901, HeLa, HGC-27, A549, EC9706, SKMES-1, KYSE450, SPC-A1, CNE1, 95D, CNE2, Jeko-1, SW620, Raji, SW480, U937, LOVO, A375, HuH-7, PLC/PRF/5 1, HFF, H22	IC_50, Average_ = 1.353 μg/mL	—	[90]
	Anticancer	Plasmid substrate, pBR32	MIC = 0.4 μM	Inhibit the binding of Topo I to DNA	[117]
	Anticancer	Human oral epidermoid carcinoma cell line KB and its vincristine-selected derivative ABCB1-overexpressing cell line KBv200, human breast carcinoma cell line MCF-7 and its doxorubicin-selected derivative ABCB1-overexpressing cell line MCF-7/Adr, human epidermoid carcinoma cell line KB-3-1 and its doxorubicin-selected derivative ABCC1-overexpressing cell line CA120, human colon carcinoma cell line S1 and its mitoxantrone-selected derivative ABCG2-overexpressing cell line S1-M1-80, human lung carcinoma cell lines A549, GLC82, and H460	IC_50_ = 0.080–0.308 μmol/L	Downregulate the expression of ABCG2 protein by activation of calpain 1, inhibit the growth of SP cells, and decrease the percentage of SP cells	[118]
	Cytotoxicity	Human colon carcinoma cell line S1, non-small-cell lung cancer cell line H460, MCF-7, and their corresponding mitoxantrone-selected derivative ABCG2-overexpressing cell lines S1-MI-80, H460/MX20, doxorubicin-selected cell line MCF-7/ADR, human normal colon epithelial cells (NCM460), and human umbilical vein endothelial cells (HUVEC)	IC_50_ = 3.8–27 μmol/L	Induce cancer cell death through c-Jun/Src/STAT3 signaling axis by inhibiting the proteasome-dependent degradation of c-Jun in both sensitive cells and ABCG2-mediated MDR cells	[119]
Secalonic acid E (**63**)	Cytotoxicity	SW-620 (colon)	IC_50_ = 19.12 μM	—	[61]
	Antibacterial	Gram-positive bacterium *M. luteus*	MIC = 36 μg/mL	—	[61]
Secalonic acid F (**64**)	Cytotoxicity	HL60 cells	IC_50_ = 4 μg/ml	Induce RhoGDI 2 differential expression, caspase 3 activation, and RhoGDI 2 cleavage	[66]
		HT1080, Cne2, and Bel7402 cell lines	IC_50_ = 11.43–16.6 μmol/L	—	[120]
	Hypoglycemic	Protein tyrosine phosphatase 1B (PTP1B)	IC_50_ = 9.6 μM	Inhibit PTP1B	[62]
Secalonic acid G (**65**)	Antibacterial	Gram-positive bacterium *M. luteus* and *S. aureus*	MIC = 5 μg/mL MIC = 39 μg/mL	—	[61]
	Cytotoxicity	MDA-MB-435 (melanoma) and SW-620 (colon) cancer cell lines	IC_50_ = 3.27 μM IC_50_ = 3.67 μM	—	[61]
Diaporxanthone F (**66**)	Antifungal	*Colletotrichum musae* (ACCC 31244)	Minimum dosages 2.5 μg/scrip	—	[68]
Dicerandrol A (**68**)	Cytotoxicity	Human breast MDA-MB-435, human colon HCT-116, human lung Calu-3, and human liver Huh7 HCT-116 and A549 cells	IC_50_ < 10 μM IC_100_ = 7.0 μg/mL	—	[71,72]
	Cytotoxicity	HepG2 cell	IC_50_ = 4.83 ± 0.22 μmol/L	—	[73,89]
	Antimicrobial	*Xanthomonas oryzae* KACC 10331, Gram-positive bacteria (*S. aureus* KCTC 1916, *B. subtilis* KCTC, *Clavibacter michiganesis* KACC 20122), yeast (*C. albicans*)	MIC = 0.125–8 μg/mL	—	[121]
	Antimicrobial	*S. aureus* and *B. subtilis*	10.8 mm and 11.0 mm (zones of inhibition resulting from 300 μg/disk)	—	[72]
Dicerandrol B (**69**)	Antimicrobial	*S. aureus* and *B. subtilis*	8.5 mm and 9.5 mm (zones of inhibition resulting from 300 μg/disk)	—	[72]
	Cytotoxicity	MDA-MB-435, HCT-116, Calu-3, and Huh7 cells HCT-116 and A549 cells	IC_50_ < 10 μM IC_100_ = 1.8 μg/mL	—	[71,72]
	Anticancer	Human cervical cancer HeLa cells	IC_50, 24h_ = 7.13 μg/mL IC_50, 48h_ = 3.00 μg/mL IC_50, 96h_ = 1.84 μg/mL	Inhibit HeLa cell viability and induce G2/M cell cycle arrest, increase the levels of GRP78, ubiquitin, cleaved PARP, and Bax protein, decrease the levels of PARP and Bcl-2 protein, increase the Bax/Bcl-2 ratio, increase the production of ROS	[13]
	Antimicrobial	*X. oryzae* KACC 10331	MIC = 16 μg/mL	—	[121]
Dicerandrol C (**70**)	Cytotoxicity	HCT-116 and A549 cells	IC_100_ = 7.0 μg/mL IC_100_ = 1.8 μg/mL	—	[72]
	Antimicrobial	*S. aureus* and *B. subtilis*	7.0 mm and 8.0 mm (zones of inhibition resulting from 300 μg/disk)	—	[72]
	Antimicrobial	*X. oryzae* KACC 10331	MIC > 16 μg/mL	—	[121]
	Anticancer	HepG2 and HeLa cancer cells	IC_50, 48h_ = 4.17 ± 0.49 μM IC_50, 48h_ = 5.18 ± 0.56 μM	Downregulate the transcription level of β-catenin-stimulated Wnt target gene and the expression of related proteins, including p-GSK3-β, β-catenin, LEF1, Axin1, c-Myc, and CyclinD1; and upregulate GSK3-β expression	[122]
Hirtusneanoside (**71**)	Antimicrobial	Gram-positive bacteria *S. aureus* and *B. subtilis*	LD_50_ = 0.0034 μM LD_50_ = 0.0140 μM	—	[75]
Chrysoxanthone H (**73**)	Cytotoxicity	H23 human non-small-cell lung carcinoma cells	IC_50_ = 6.9 μM	—	[59]
Versixanthone G (**74**)	Anticancer	Human leukemia cell lines HL-60 and K562, lung cancer cell line A549, non-small-cell lung cancer cell H1975, human gastric cancer cell line MGC803, human embryonic kidney HEK293, human ovarian carcinoma cell line HO-8910, and human colon cancer cell line HCT-116	IC_50_ = 4.6–20.9 μM	Inhibit Topo I, arresting the cell cycle at the G2/M phase	[76]
Versixanthone H (**75**)	Anticancer	HL-60, K562, A549, H1975, MGC803, HEK293, HO-8910, HCT-116	IC_50_ = 5.3–22.1 μM	Inhibit Topo I	[76]
Ascherxanthone A (**76**)	Antiparasitic	*P. falciparum* K1	IC_50_ = 0.20 μg/mL	—	[77]
	Cytotoxicity	African green monkey kidney fibroblast (Vero) Human epidermoid carcinoma cells (KB) Human breast cancer cells (BC) Human lung cancer cells (NCI-H187)	IC_50_ = 0.16–1.7 μg/mL	—	[77]
Ascherxanthone B (**76**)	Antifungal	*Magnaporthe grisea*	IC_90_ = 0.58 μg/mL	—	[78]
Muyocoxanthone F (**79**)	Anti-inflammation	LPS-stimulated RAW264.7 cells	IC_50_ = 1.3 μM	Inhibit production of NO	[79]
Rugulotrosin A (**80**)	Antibacterial	Gram-positive (*Enterococcus faecalis*, *B. cereus*, *S. aureus*)	LD_99_ = 1.6 μg/mL LD_99_ = 3.1 μg/mL LD_99_ = 200 μg/mL	—	[80]
Xanthonol (**81**)	Insecticidal and anthelmintic activities	*Lucilia sericata*, *Aedes aegypti*, *Haemonchus contortus*	LD_99_ = 33 μg/mL LD_99_ = 8 μg/mL LD_99_ = 50 μg/mL	—	[81]
Penicixanthone G (**87**)	Cytotoxicity	Human carcinoma A549, HeLa, and HepG2 cells	IC_50_ = 0.3–0.6 μM	—	[84]
	Antibacterial	*S. aureus* and the methicillin-resistant strain MRSA	MIC = 0.4 μg/mL	—	[84]
Aflaxanthone A (**89**)	Antibacterial	Pathogenic bacteria (methicillin-resistant *S. aureus* A7983, *B. subtilis* ATCC 6633)	MIC = 12.5 μM MIC = 25 μM	—	[18]
	Antifungal	*C. albicans* ATCC 10231 *Fusarium oxysporum* *Penicillium italicum* *Collettrichum musae* *Colletotrichum gloeosporioides*	MIC = 12.5 μM MIC = 12.5 μM MIC = 50 μM MIC = 25 μM MIC = 3.13 μM	—	[18]
Aflaxanthone B (**90**)	Antibacterial	*B. subtilis* ATCC 6633	MIC = 25 μM	—	[18]
	Antifungal	*C. albicans* ATCC 10231, *F. oxysporum*, *C. musae*, and *C. gloeosporioide*	MIC = 12.5–25 μM	—	[18]
Versixanthone M (**92**)	Cytotoxicity	HL-60, K562, A549, H1975, MGC803, HO-8910, HCT-116	IC_50_ = 0.4–11.7 μM	—	[76]
Chrysoxanthone K (**93**)	Cytotoxicity	H23 human non-small-cell lung carcinoma cells	IC_50_ = 3.9 μM	—	[59]
Phomoxanthone A (**94**)	Cytotoxicity	Two cancer cell lines (KB, BC-1) and Vero cells	IC_50_ = 0.51–1.4 μg/mL	—	[87]
	Antitubercular	*Mycobacterium tuberculosis* (H37Ra strain)	IC_50_ = 0.5 μg/mL	—	[87]
	Antimalarial	*P. falciparum* (K1, multidrug-resistant strain)	IC_50_ = 0.11 μg/mL	—	[87]
	Antimicrobial	*B. subtilis*	MIC = 7.81 μg/mL	—	[74]
12-*O*-Deacetylphomoxanthone A (**96**)	Cytotoxicity	HepG2, HeLa cells	IC_50_ = 12.06 ± 0.55 μM IC_50_ = 20.36 ± 1.99 μM	—	[89]
	Anticancer	Human ovarian cancer (OC) cells (SKOV-3 and ES-2)	IC_50_ = 6.08 μM IC_50_ = 3.8 μM	Downregulate PDK4	[123]
4-4’-secalonic acid D (**99**)	Cytotoxicity	BGC-823, SKHEP, SGC-7901, HeLa, HGC-27, A549, EC9706, SKMES-1, KYSE450, SPC-A1, CNE1, 95D, CNE2, Jeko-1, SW620, Raji, SW480, U937, LOVO, A375, HuH-7, PLC/PRF/5 1, HFF, H22	IC_50, Average_ = 1.026 μg/mL	—	[90]
Chrysoxanthone J (**100**)	Cytotoxicity	H23 human non-small-cell lung carcinoma cells	IC_50_ = 6.4 μM	—	[59]
Penicillixanthone A (**101**)	Anti-HIV-1	TZM-bl cells CCR5-tropic HIV-1 SF162 and CXCR4-tropic HIV-1 NL4-3	IC_50_ = 0.36 μM IC_50_ = 0.26 μM	—	[91]
	Cytotoxicity	MDA-MB-435 (melanoma) and SW-620 (colon) cancer cell lines	IC_50_ = 0.18 μM IC_50_ = 0.21 μM	—	[61]
	Antimicrobial	Gram-positive bacterium *M. luteus* and *S. aureus*	MIC = 46 μg/mL MIC = 93 μg/mL	—	[61]
	Antibacterial	*S. aureus*	MIC = 6.25 μg/mL	—	[17]
Penicillixanthone B (**102**)	Antimicrobial	Gram-positive bacterium *M. luteus* and *S. aureus*	MIC = 15 μg/mL MIC = 59 μg/mL	—	[61]
	Cytotoxicity	MDA-MB-435 (melanoma) and SW-620 (colon) cancer cell lines	IC_50_ = 5.20 μM IC_50_ = 5.55 μM	—	[61]
Secalonic acid F1 (**103**)	Antibacterial	*S. aureus*	MIC = 25 μg/mL	—	[17]
Deacetylphomoxanthone B (**104**)	Antimicrobial	*X. oryzae* KACC 10331	MIC = 4 μg/mL	—	[121]
	Cytotoxicity	MDA-MB-435, HCT-116, Calu-3, and Huh7 cells	IC_50_ < 10 μM	—	[71]
Phomoxanthone B (**105**)	Cytotoxicity	MCF7 cells A549 cells 830 cells	IC_50, 72h_ = 0.45 ± 0.12 μM IC_50, 72h_ = 8.01 ± 0.15 μM IC_50, 72h_ = 2.96 ± 1.04 μM	Induce apoptosis, cell cycle arrest at the G2/M phase, inhibit the migration and invasion	[92]
	Cytotoxicity	Two cancer cell lines (KB, BC-1) and Vero cells	IC_50_ = 0.7–4.1 μg/mL	—	[87]
	Antimalarial	*P. falciparum* (K1, multidrug-resistant strain)	IC_50_ = 0.33 μg/mL	—	[87]
	Antitubercular	*M. tuberculosis* (H37Ra strain)	MIC = 6.25 μg/mL	—	[87]
Penexanthone A (**106**)	Cytotoxicity	MDA-MB-435, HCT-116, Calu-3, and Huh7 cells	IC_50_ < 10 μM	—	[71]
	Anticancer	Human colorectal cancer cells (HCT116, SKOV3, MCF-7, A549, and PC3)	IC_50_ = 1.27–7.08 μg/mL	Enhance the sensitivity of colorectal cancer (CRC) to CDDP and induce ferroptosis by targeting Nrf2 inhibition	[124]
Versixanthone J (**107**)	Cytotoxicity	HL-60 cells	IC_50_ = 47.3 μM	—	[76]
Versixanthone K (**108**)	Anticancer	HO-8910, HEK293 cells	IC_50_ = 49.5 μM	Inhibit Topo I	[76]
Versixanthone L (**109**)	Cytotoxicity	HL-60, K562, A549, MGC803, HO-8910, HCT-116 cells	IC_50_ = 0.5–1.6 μM	—	[76]
Neosartorin (**110**)	Antibacterial	MRSA MB5393	MIC = 128 μg/mL	—	[94]
Deacetylneosartorin (**111**)	Antibacterial	MRSA	MIC = 64 μg/mL	—	[94]
Rugulotrosin B (**113**)	Antibacterial	*B. subtilis*	LD_99_ = 25 μg/mL	—	[80]
Eumitrin H (**115**)	Antibacterial	*E. coli* ATCC25922, *Pseudomonas aeruginosa* ATCC27853, *S. aureus* ATCC25923, *C. albicans* TISTR	IC_50_ = 125–250 μg/mL		[95]
	Antitumor	Tyrosinase	IC_50_ > 200 μM	Inhibit tyrosinase	[95]
	Hypoglycemic	α-glucosidase	IC_50_ = 64.2 ± 0.51 μM	Inhibit α-glucosidase	[95]
Asperdichrome (**116**)	Hypoglycemic	PTP1B	IC_50_ = 6.0 μM	Inhibit PTP1B	[62]
	Antibacterial	*S. aureus*	MIC = 25 μg/mL		[17]
Eumitrin C (**120**)	Antiparasitic	*P. falciparum* FcB1	IC_50_ = 96.5 ± 3.5 μM	—	[96]
Subplenone H (**123**)	Antibacterial	Methicillin-resistant *S. aureus* ATCC 43300 and 700698, *S. aureus* ATCC 29213 MSSA, vancomycin-resistant *E. faecalis* ATCC 51299, vancomycin-resistant *E. faecium* ATCC 700221, *E. faecalis* ATCC 29212 VSE, and *S. epidermidis* ATCC 12228 MSSE	MIC = 0.125–2.0 μg/mL	—	[56]
Nidulaxanthone A (**129**)	Cytotoxicity	HepG2 SW1990, MCF-7, HCT116, and LO2 cells	IC_50_ = 21.9 μM IC_50_ > 30 μM	—	[98]
Eumitrin F (**130**)	Antibacterial Antitumor	*E. coli* ATCC25922, *P. aeruginosa* ATCC27853, *S. aureus* ATCC25923, *B. subtilis* ATCC6633, *C. albicans* TISTR	IC_50_ = 62.5–500 μM	—	[95]
Eumitrin G (**131**)	Antibacterial	As above	IC_50_ = 62.5–500 μM	—	[95]
Phomoxanthone D (**133**)	Immunosuppressive	The proliferation of ConA-induced (T-cell) and LPS-induced (B-cell) murine splenic lymphocytes	IC_50_ = 44.84 ± 1.26 μM IC_50_ = 77.76 ± 1.47 μM	—	[89]
Cladoxanthone B (**134**)	Cytotoxicity	MB49 (sensitive mouse bladder carcinoma cells), J82 (human bladder carcinoma cells), 4T1 (mouse breast carcinoma cells), and Huh7 (human hepatocellular carcinoma cells)	IC_50_ = 24.7–46.4 μM	—	[99]
Ergoflavin (**136**)	Anti-inflammation	Human TNF-a and IL-6 THP-1 cells and human PBMCs	IC_50_ = 1.9 ± 0.1 μM IC_50_ = 1.2 ± 0.3 μM	Inhibit TNF-α and IL-6	[100]
	Cytotoxicity	ACHN, H460, Panc1, HCT116, and Calu1 cancer cell lines	IC_50_ = 1.0 –8. 45 μM	—	[100]
Swertifrancheside (**138**)	Antiviral	Inhibitor of HIV reverse transcriptase	Inhibitory activity of 99.8% at 200 μg/mL (ED_50_ = 30.9 μg/mL)	—	[33]
Subplenone J (**154**)	Antibacterial	Methicillin-resistant *S. aureus* ATCC 43300 and 700698, *S. aureus* ATCC 29213 MSSA, vancomycin-resistant *E. faecalis* ATCC 51299, vancomycin-resistant *E. faecium* ATCC 700221, *E. faecalis* ATCC 29212 VSE, and *S. epidermidis* ATCC 12228 MSSE	MIC = 0.125–4.0 μg/mL	—	[56]
Secalonic acid H (**155**)	Antibacterial	*S. aureus*	MIC = 25 μg/mL	—	[17]
Versixanthone I (**156**)	Cytotoxicity	HL-60 cells	IC_50_ = 27.8 μM	—	[76]
Chrysoxanthone (**158**)	Antimicrobial	*Arthrobacter citreus*, *Penicillium notatum* *Bacillus brevis*, *Corynebacterium insidiosum*, *Aspergillus ochraceus*	MIC = 2.5–20 μg/mL	—	[105]
	Cytotoxicity	Jurkat, L-1210, Colo-320, and HeLa-S3 cells	IC_50_ > 50 μg/mL	—	[105]
Chrysoxanthone C (**160**)	Antibacterial	*S. aureus*	MIC = 50 μg/mL	—	[17]
Muyocoxanthone K (**166**)	Anti-inflammation	LPS-stimulated RAW264.7 cells	IC_50_ = 5.1 μM	Inhibit production of NO	[79]
Versixanthone D (**167**)	Cytotoxicity	HL-60, K562, A549, H1975, 803, HO8910, and HCT-116 cells	IC_50_ = 3.1–13.9 μM	—	[16]
Versixanthone F (**168**)	Cytotoxicity	HL-60, K562, A549, HO8910, and HCT-116 cells	IC_50_ = 0.7–20.8 μM	—	[16]
Diaporxanthone A (**170**)	Antifungal	*Nectria* sp.	Minimum dosages 10 μg/scrip	—	[68]
Diaporxanthone D (**174**)	Cytotoxicity	A2870 human ovarian cancer, HepG2 human liver cancer, EC109 human esophagus cancer, PC3 human prostate cancer, A549 human lung adenocarcinoma cancer cell lines, and HBE human bronchial epithelial cell lines	IC_50_ = 1.66–8.4 μM	—	[68]
Versixanthone B (**175**)	Cytotoxicity	HL-60, 803 cells	IC_50_ = 9.9–21.6 μM	—	[16]
Versixanthone C (**178**)	Cytotoxicity	HL-60, K562, H1975 cells	IC_50_ = 7.8–25.6 μM	—	[16]
Versixanthone A (**185**)	Cytotoxicity	HL-60, K562, H1975, HO8910 cells	IC_50_ = 2.6–11.2 μM	—	[16]
Versixanthone E (**186**)	Cytotoxicity	HL-60, K562, H1975, 803, HO8910 cells	IC_50_ = 1.6–11.1 μM	—	[16]
	Anticancer	Topoisomerase I	—	Inhibit Topo I	[16]
Phomoxanthone N (**189**)	Immunosuppressive	The proliferation of ConA-induced (T-cell) and LPS-induced (B-cell) murine splenic lymphocytes	IC_50_ = 75.75 ± 1.78 μM IC_50_ = 102.65 ± 1.38 μM	—	[89]
Phomoxanthone L (**191**)	Immunosuppressive	As above	IC_50_ = 55.53 ± 0.93 μM IC_50_ = 89.27 ± 2.25 μM	—	[89]
Phomoxanthone M (**192**)	Immunosuppressive	As above	IC_50_ = 60.25 ± 1.58 μM IC_50_ = 87.66 ± 2.76 μM	—	[89]
Eumitrin D (**194**)	Antiparasitic	*P. falciparum* FcB1	IC_50_ = 73.0 ± 1.0 μM	—	[96]
	Cytotoxicity	Huh7 (differential hepatocellular carcinoma), Caco 2 (differentiating colorectal adenocarcinoma), PC-3 (prostate carcinoma), MCF-7 cells	IC_50_ = 35 μM IC_50_ = 44 μM IC_50_ = 42 μM IC_50_ = 12 μM	—	[96]
Phomopsis-H76 A (**197**)	Proangiogenic	Zebrafish embryos	—	Accelerated the growth of sub-intestinal vessel plexus (SIV) branch markedly	[109]
Muyocoxanthone B (**201**)	Anti-inflammation	LPS-stimulated RAW264.7 cells	IC_50_ = 5.2 μM	Inhibit production of NO	[79]
Monodictyochrome B (**203**)	Anticancer	Cyclooxygenase-1 (COX-1) and aromatase enzymatic	IC_50_ = 7.5 μM	Inhibit cytochrome P450 enzymes	[110]
Monodictyochrome A (**212**)	Anticancer	As above	IC_50_ = 5.3 μM	As above	[110]

## Data Availability

Data sharing is not applicable to this article as no new data were created or analyzed in this study.
